# Ancient DNA reconstruction of Late Holocene ecosystems within the Carpathian Basin from paleo-meanders and archaeological deposits

**DOI:** 10.1038/s41598-026-35509-2

**Published:** 2026-02-03

**Authors:** Giulia Zampirolo, Anthony H. Ruter, Ivana Živaljević, Kristina Penezić, Philip Francis Thomsen, David Orton, Milica Kašanin Grubin, Nevena Antić, Mikkel Winther Pedersen

**Affiliations:** 1 Section for Molecular Ecology and Evolution, Faculty of Health and Medical Sciences, Globe Institute, University of Copenhagen, Copenhagen, Denmark; 2 Centre for Ancient Environmental Genomics, Faculty of Health and Medical Sciences, Globe Institute, University of Copenhagen, Copenhagen, Denmark; 3 Department of History, Faculty of Philosophy, University of Novi Sad, Novi Sad , Serbia; 4 BioSense Institute, University of Novi Sad, Novi Sad , Serbia; 5 Department of Biology, Aarhus University, Aarhus, Denmark; 6 BioArCh, Department of Archaeology, University of York, York, UK; 7 Institute of Chemistry, Technology and Metallurgy, University of Belgrade, Belgrade, Serbia

**Keywords:** Ecology, Ecology, Evolution

## Abstract

The diverse ecology in the Carpathian Basin supported a variety of subsistence strategies throughout the Holocene, ranging from animal husbandry to the management of woodlands, grasslands, and wetlands. The earliest evidence of aquatic resource use in the region dates back to the Early Neolithic (~ 6200−5300 cal BC), with cultural practices extending into recent times. This is reflected in the numerous open-air archaeological sites widely distributed across the landscape and on the Danube meanders. However, the archaeological record, especially organic remains and fossils, may not fully capture the region’s species diversity due to degradation and scarcity, hindering the accurate reconstruction of past human-environment interactions. Recent studies have shown that ancient DNA in sediments (*seda*DNA) can survive even in the absence of visible fossils, however, these studies have primarily been limited to cave-, lake- and permanently frozen sedimentary deposits. Here, we investigate two open-air archaeological sites and three paleo meander deposits of the Danube in Serbia (southeastern Europe) using *seda*DNA. Our analysis reveals genetic traces from past freshwater and terrestrial ecosystems and local exploitation of the migratory sturgeon species, currently extirpated from the region. We find *seda*DNA preserved in sediments from unsheltered archaeological contexts and explore the associated challenges that require further investigation.

**Supplementary Information:**

The online version contains supplementary material available at 10.1038/s41598-026-35509-2.

## Introduction

**Fig. 1:**
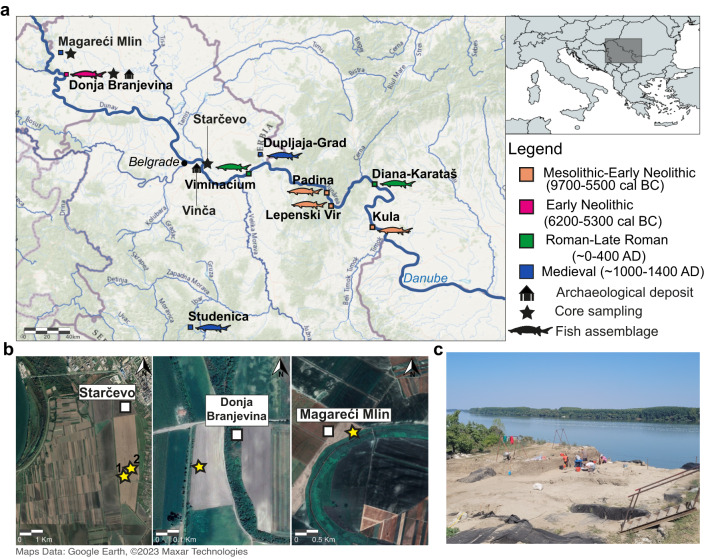
Overview of the sampling location. (**a**). Map showing the coring locations (star), the archaeological sites subsampled for DNA (house outline) with indication of sites yielding sturgeon remains^22^ (left map: ArcGIS^®^ software by Esri, July 2023, https://www.arcgis.com/index.html, sources: USGS, Esri Romania, HERE, Garmin, FAO, NOAA, USGS and World Hydro Reference Overlay; right map: mapchart.*net*). (**b**). Satellite imagery of the core locations (yellow star) and the archaeological sites (white square) at Starčevo-Grad, Donja Branjevina, and Magareći Mlin. **c**. The archaeological excavation at Vinča-Belo brdo (2021) on the Danube terrace.

The Danubian floodplain represents a dynamic environment where variations in water flow can alter the river’s trajectory, leading to the isolation of meanders that subsequently transform into oxbow lakes. These stable basins can gradually accumulate fine sediments from recurring floods or erosion of organic material from riverbanks, eventually evolving into marshes or meadows^1,2^, affecting water and land management strategies^3^. The landscape underwent major modifications with the arrival of the first farmers from Anatolia and the Aegean in the southern parts of the Carpathian Basin during the Early Neolithic (~ 6200 cal BC)^4–7^ and continued throughout the Holocene, as suggested by the archaeobotanical evidence^8–10^. Similarly, this region has provided evidence of the continuous exploitation of freshwater resources starting from this period^11–13^. A large number of archaeological sites from Early Neolithic (~ 6200−5300 cal BC), Roman (~ 0−400 AD), and Medieval (~ 1000–1400 AD) periods, located along the Serbian section of the Danube River, have yielded fish assemblages (Fig. 1a) alongside evidence of domesticated animals^14–22^, thus indicating the continued inclusion of aquatic resources into the human diet. However, it remains a challenge to accurately investigate the role of humans in exploiting the environment, particularly when relying solely on fossil evidence. Small and degraded remains, such as fish bones, are difficult to identify and are typically recovered in low quantities. For example, the high abundance of domestic animal (mammal) remains compared to the smaller proportion of fish bones, particularly sturgeon, at the Neolithic sites of Starčevo-Grad and Donja Branjevina (Serbia) may be a result of taphonomic processes affecting sturgeon cartilaginous skeleton and consequent collection biases, rather than their actual absence from the diet^15,23^.

Preliminary surveys^24,25^ of archaeological sites in the area of the middle Danube have revealed old meanders dated to the late and early Holocene and currently buried beneath younger alluvium^24^. These fluvial contexts and the associated archaeological layers can be defined as “open-air deposits”, which refers to natural or anthropogenic accumulations of sediment subjected to increased environmental fluctuations, in contrast to those enclosed in caves, rock shelters, or submerged environments such as lake sediments.

Recent studies have highlighted the potential of sedimentary ancient DNA (*seda*DNA) to identify the biological components of the environment on a temporal scale^26,27^. Most of the *seda*DNA applications have focused on lake deposits^28–30^ and permafrost^31–33^ which offered valuable insights into climatic and ecosystem changes. Additionally, caves^34–37^ and rock shelters^38^ have served as unique archives for studying human history and faunal extinctions. Only a few studies have directly investigated open-air archaeological contexts^39–44^, while paleo-meander deposits remain unexplored. This is primarily due to the significant challenges associated with unsheltered deposits, such as weathering and erosion, which can affect DNA preservation and alter the depositional context.

In this study, we investigate the preservation of *seda*DNA in sediments from two different open-air archaeological sites in Serbia, (i) the Neolithic site of Donja Branjevina^11,25^, and (ii) the Late Neolithic site of Vinča-Belo Brdo^45,46^. In addition, we collected four sediment cores from paleo-meanders in proximity to the sites of Starčevo-Grad (two cores)^47^, Donja Branjevina, and Magareći Mlin^48^, all located in the middle Danube basin (Serbia). We find genetic evidence of past terrestrial and freshwater ecosystems and we assess the changes in human land use on a time scale spanning the early and late Holocene. Our results demonstrate both the potential and the challenges of applying *seda*DNA to ancient deposits found in open-air sites.

## Methods

### Sampling sedimentary deposits

Sediments were collected during the 2021 and 2022 excavations of Donja Branjevina (West Bačka District, Vojvodina province, Serbia) and Vinča-Belo brdo (Grocka, Belgrade, Serbia) sites. Both sites are located on alluvial terraces and are composed primarily of silt and sand, intermixed with organic matter and anthropogenic material^25,49^. Samples were taken from five layers at Donja Branjevina, covering 5 units (Figure S2.1b) corresponding to the Neolithic occupation (~ 6100−5600 cal BC), and from six layers (73–100 cm), at Vinča, representing three phases of the Late Neolithic (~ 4550 cal BC) house nr. 1/20^46^ (Figure S2.1a). The exposed profiles were first cleaned by removing about 10 cm of sediments using a sterilised trowel and scalpels. Samples were then collected starting from the bottom of the cleaned profile to reduce the risk of introducing materials from higher, more recent layers and using a sterile disposable scalpel while wearing sleeves, face masks and nitrile gloves.

In the spring of 2022, four cores were drilled for 2 m depth from paleo meanders in the middle Danube basin (Serbia), following the traces of former river channels evident in the terrain and on satellite imagery (Fig. 1b). These are situated near archaeological deposits from the Early, Middle, and Late Holocene and in rural areas occupied by agriculture (Table S2.2). The collection was conducted in collaboration with the Institute of Field and Vegetable Crops (Novi Sad, Serbia) and the BioSense Institute (Novi Sad, Serbia) using a soil column cylinder auger set (1 m length, 90 mm diameter) powered by a gasoline percussion hammer (Cobra TT). All cores collected were transferred into 1-meter PVC tubes, and securely sealed immediately upon collection at the field site. All samples were hereafter transported to the ancient DNA dedicated laboratories at the Globe Institute, University of Copenhagen (Denmark), the sediment samples were stored at -20° C until DNA extraction, while sediment cores were kept at ~ 6° C until further subsampling.

### Sedimentology and DNA subsampling of the paleo-channels

The four cores were subsampled for DNA and grain size analysis: the two cores at Starčevo (abbreviated “STR1” and “STR2”), core 2 at Donja Branjevina (abbreviated “DOB2”), and core 1 at Magareći Mlin (abbreviated “MML1”). We proceeded with core subsampling in a dedicated room at the Globe Institute, University of Copenhagen, wearing full protective suits, gloves, and face masks, and treating all equipment and surfaces with bleach and ethanol to minimise the risk of contamination. The surface sediment was removed with sterilised scalpels and samples were taken from the centre of the core every 5–10 cm using sterilised syringes, for a volume of ca. 5 ml. Intervals of 2 cm were used at the bottom of core DOB2, to cover the numerous inclusions in the sandy layer. The samples for the DNA analysis were then frozen at -20 °C.

Sediment texture was described using the soil ribbon test by screening it through nested sieves (500 μm, 200 μm, 70 μm) using The United States Department of Agriculture (USDA) soil texture classes^50^. The relative proportions of coarse and fine fractions (≥ 63 μm and < 63 μm) were analysed at the Institute of Technology and Metallurgy, University of Belgrade, Belgrade, Serbia (Dataset S1). The 500 μm screened fraction was inspected for macrofossils using an Olympus SZX7 stereomicroscope (5x–56x magnification) and photo documented with a Canon EOS RP 26.2 megapixel camera. Plant macrofossils were identified using the seed reference collection at Globe Institute, University of Copenhagen, and several published keys^51,52^. To estimate the organic carbon (OC) and calcium carbonate (CaCO3) content, all alluvial sequences were subsampled at a 5 cm interval for loss on ignition (LOI) following the protocols of Dean (1974)^53^ and Heiri et al. (2001)^54^ with some minor modifications.

### Radiocarbon dating

A total of 23 plant macrofossils were radiocarbon dated (Table S2.1) to establish a chronology within each of the cores. Where possible, we dated the top and bottom of the lithological section. The AMS dates were processed at the Higham lab, Department of Evolutionary Anthropology, University of Vienna, and Beta Analytic (for laboratory codes, and ages see Table S2.1). All dates were calibrated using IntCal20^55^ and OxCal 4.4.4.^56^, except those with very recent or modern conventional ages. These were calibrated using the NH1 Bomb curve of INTCAL20 calibration curve compendium^55^.

### DNA extraction and library preparation

A total of 51 sediment samples were processed for DNA extraction and library preparation at the Centre for Ancient Environmental Genomics, University of Copenhagen. DNA extraction was performed using MagAttract PowerSoil DNA KF Kit (Qiagen) following manufacturer’s instructions. The samples from the archaeological sites of Donja Branjevina and Vinča- Belo brdo were separately extracted using a lysis buffer and a phenol-chloroform purification protocol as described in Pedersen et al.^29^. including a total of 2 extraction and 2 library negative controls. Double-stranded Illumina libraries were prepared following Meyer and Kircher (2010)^57^. Amplification cycles were determined empirically using a qPCR, and the chosen number of cycles given to each library was the cutting threshold (ct) value −2 cycles (between 12 and 19 cycles). Unique indexes were added to each library in a 25ul volume PCR reaction containing 10.5 ul KAPA HiFi Uracil + mastermix (Roche) with 0.8 μm of each forward and reverse primer added to 10.5 ul of the library. We then purified the libraries with magnetic beads (MagBio HighPrep PCR, MagBio Genomics Inc., USA) at a 1:1.8 ratio. All libraries were equimolarly pooled and sequenced on a NovaSeq 6000 150, paired-end (GeoGenetics Sequencing Core, University of Copenhagen).

### Metagenomic analysis

We sequenced a total of 10,278,821,887 reads, which were trimmed and collapsed with default settings using AdapterRemoval (v2.3.0)^58^. A total of 2,996,716,628 collapsed reads were hereafter processed through the ‘Holi’ pipeline^34^ to remove low complexity reads, reads below = < 30 bp and duplicates (Figures S3.1, S3.2). All reads were hereafter competitively mapped against publicly accessible databases, including the NCBI non-redundant nucleotide (nt) database and the RefSeq database (release 213) using Bowtie2 (v. 2.3.2)^59^. To mitigate erroneous alignments of bacterial-like sequences to eukaryotic references^60^, we incorporated the Genome Taxonomy Database^61^ (GTDB, release 202) into the competitive mapping. We additionally complemented our genome databases with the reference genome assemblies of the Russian sturgeon (*Acipenser gueldenstaedtii *(A recent publication^62^ suggested reassigning the *Acipenser* species to the genus *Huso*. As this revised classification is not yet widely accepted, we use the conventional genus names throughout this study, GCA_024762115.1) and beluga (*Huso huso*, GCA_036884735.1) to enhance the detection of sturgeon DNA in our dataset, as they were not part of the RefSeq database. Next, all alignments were merged and sorted by coordinate using samtools^63^ and parsed through metaDMG (version 0.38)^64^ for taxonomic classification (*--min-similarity-score* 0.95 *--max-similarity-score* 1.0 *--damage-mode* lca) and damage estimation, using a modified NCBI taxonomy file that includes taxids and accessions for each contig or chromosome of the additional reference sequences. We filtered the output damage statistics following recommendations in Michelsen et al. (2022)^64^ and employed a data-driven approach, requiring each family to have a minimum of 100 reads and a significance of 2. To minimise the risk of overmatching due to incomplete reference genomes for the genera in the study area^65^, we conservatively present all results at the taxonomic level of family. Lastly, we built a model based on the amplitude of damage (*MAP_damage*) limited to terrestrial plants, restricting to taxa with > = 500 reads and a significance > 2 (Figures S4.1-S4.4). We used the minimum values identified in this model to filter the remaining dataset with read counts down to 100 in order to ensure all taxa followed the assumption that DNA damage increases with age similar to the observation by the terrestrial plant data^66^ (see Figures S4.5-S4.13 for further details on the filtered taxa). The filtered reads from Donja Branjevina and Magareći Mlin cores were plotted to a composite diagram using Psimpoll^67^ and zoned with CONIIC, an agglomerative constrained cluster analysis, using the information content statistic to form the initial dissimilarity matrix^68^. Only the plant reads were used to determine the zones. All main figures were edited in Adobe Illustrator (v. 24.0.3).

## Results

### Paleo-meanders: sedimentology and chronology

Grain size and sediment textural analysis from the four cores reveal that both Magareći Mlin and Donja Branjevina are discontinuous alluvial sequences derived from multiple sporadic sedimentation events (Tables S1.1, S1.2, and Figure S1.1a-d). Seasonal floods and erosion events driven by water movement have significantly impacted the deposition and sources of sediments and macrofossils. At the Donja Branjevina site, this process has resulted in a complex chronology, with Early Neolithic ages (~ 5905−4700 cal BC) occurring as outliers within the lower section of the core (Z1), on top of the Late Iron Age (~ 288−281 cal BC). The presence of sand in this zone suggests moderate energy river flow, which likely caused erosion of the riverbanks and the incorporation of older materials into the sedimentary record. Similarly, the upper section (Z2) contains sediments from both the Middle Ages (~ 1330 cal AD) and the 18th century (~ 1770 cal AD), also inversely stratified (Fig. 3). Here, the layer is composed of fine sandy loam, which is indicative of a low-energy channel fill. The three clay layers at the top of the sequence are likely low-energy over-bank deposits (Z3-5), which, given the modern ages (~ 1900 cal AD), may be associated with flood events from a modern channel near the sampling site (Fig. 1b).

In contrast, the sediments at Magareći Mlin exhibit a more stable accumulation rate, spanning from the Iron Age (~ 751−419 cal BC) to modern times (~ 1950 cal AD). Evidence of high-energy river flow is limited to Zone 1 (175–200 cm), and the majority of the sequence is characterised by clay rich in organic carbon (Figure S1.1d, e). This stratigraphic structure is frequently observed in fluvial sediments from floodplains, where variations in the water flow can lead to the abandonment of meanders, which results in a lower-energy water basin (also called an oxbow lake). Under these conditions, finer particles gradually accumulate, while coarser sediments are preferentially deposited along the channels of the active meander. The low concentration of calcium carbonate (mean = 4%) compared to the other sites overall (mean = 16%) (Figure S1.1e) also supports this interpretation. Additionally, the presence of bone fragments (Table S1.2, Figure S1.2) might suggest the incorporation of material from the nearby archaeological tell into the sediment. The uppermost layer consists of clayey alluvium, dating to the recent period (~ 1980 AD), and likely represents over-bank deposition from the active channel near the sampling location, like the upper layers observed at Donja Branjevina (Fig. 1b). In contrast, macrofossil remains were scarce at Starčevo-Grad, which hindered the construction of a continuous chronology for this site (Table S2.1). The two cores (STR1 and STR2) can be correlated based on the sedimentology (Table S1.1 and Figure S1.1a, b), composed primarily of sand and silt with grey clay inclusions. The earliest date is identified from the bottom section of core STR1, corresponding to the Middle Ages (~ 1164–1220 cal AD), while the mid-upper sections of both Starčevo cores cover the period between the 18th and the 21st centuries (Table S2.1).

### Taxonomic profiling and DNA damage estimation

A total of 3,012,071,419 reads passed quality control for duplicates and low-complexity sequences and were therefore mapped and then parsed through metaDMG (v.0.38) for taxonomic profiling and post-mortem DNA damage estimation^64^ (see Methods for further details). We built a DNA damage-age model using the frequency of C to T transitions observed on the first position (*MAP_damage*) for all identified terrestrial plants with 500 unique reads or more (Figures S4.1-S4.4). We used the minimum degree of damage from this model to filter all identified taxa, which we visualise at the family level along with the mean fragment length by depth in Figures S4.6-S4.13. Overall, we find that ancient DNA is recovered throughout both sequences at Magareći Mlin and Donja Branjevina, and with a higher proportion of reads classified to plants compared to animals (Figure S4.5b, d). In both locations, DNA damage varies between 5 and 15% in the upper layers and between 20 and 50% in the middle layers, except for a lower percentage of damage (~ 10–25%) found at the bottom of both cores in layers that consisted of coarse sediments. The DNA found in the archaeological layers in the different sites reveals that the plants showed up to 40% damage at the site of Donja Branjevina and 30% at Vinča-Belo brdo (Figures S4.6, S4.7), and that the abundance of animal DNA was generally low (reads < 100), similar to the observation in the paleo-meanders. At Starčevo-Grad, we found limited detection of plant and animal DNA reads exhibiting damage patterns (Figures S4.5e, f; S4.12, S4.13), along with difficulties in establishing a defined chronology, which precluded any further analysis of these cores and data.

### Plant DNA in the archaeological deposits

**Fig. 2:**
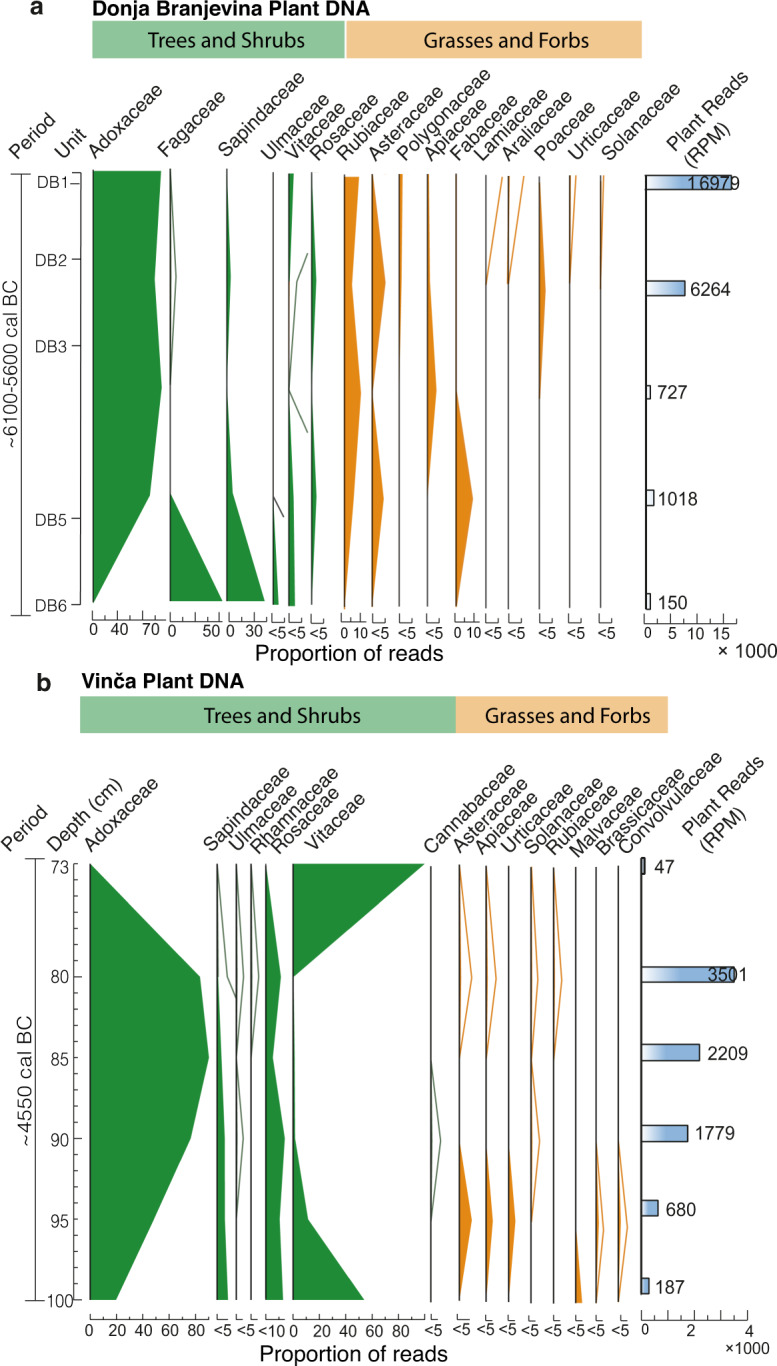
Plant DNA from archaeological deposits. Stratigraphic plots showing the proportion of ancient reads for plant DNA detected at the sites of Donja Branjevina (**a**) and Vinča Belo-brdo (**b**). The total plant read counts are displayed on the right, reported as reads per million (RPM).

We sampled the Late Neolithic deposits at Vinča-Belo Brdo (~ 4700/4600 cal BC)^45,69^ and the Neolithic contexts at Donja Branjevina (~ 6100−5600 cal BC)^25^ to assess DNA preservation at an open-air archaeological site. The vegetation identified (Fig. 2) corresponds to the mid-Holocene mixed-deciduous forest typical of the northern-central Balkans, as also described by ^10^. Woody taxa include Sapindaceae and Ulmaceae, most likely representing maple (*Acer*), elms (*Ulmus*), and horse chestnut (*Aesculus*), which are characteristic of the Vojvodina region during the period 5000−3000 BC. Fagaceae is exclusively observed at Donja Branjevina, likely representing *Quercus* (oak), known to have been widespread throughout the region during the Neolithic^10^.

At both sites, we find families that include herbs and shrubs typically bearing edible fruits, such as elderberry (*Sambucus*, Adoxaceae) and the cranberry bush (*Viburnum sp.*, Adoxaceae), roses/plums (Rosaceae), and grapes (Vitaceae), all of which represent potential food sources. Our findings align with archaeobotanical remains from the site of Vinča-Belo brdo and from the Neolithic settlements of Gomolava (~ 4800−4600 cal BC), Opovo (~ 4900−4700 cal BC), and Starčevo (~ 5800−5000 BC), where wild pear (*Pyrus* sp., Rosaceae), elderberry (*Sambucus*, Adoxaceae), apple (*Malus* sp., Rosaceae), and grapes (*Vitis Vinifera*, Vitaceae) were similarly identified^10,70–73^.

In addition to these taxa, we also detect families associated with open habitats. At Donja Branjevina, we observe grasses (Poaceae), legumes (Fabaceae), and Polygonaceae, which includes many disturbance-adapted ruderal genera. At Vinča-Belo Brdo, we find Malvaceae, which may be derived from either the common lime, *Tilia* sp., or possibly more likely mallow, *Malva* sp., given that it co-occurs with nightshades (Solanaceae) and bindweeds (Convolvulaceae) typical of disturbed substrates. The presence of forbs and shrubs (Asteraceae) at both sites also suggests open or semi-open habitats. At Donja Branjevina, their co-occurrence with grasses (Poaceae) and the Polygonaceae is also indicative of mainly open landscapes. These may reflect anthropogenic disturbance, primarily due to grazing, but these families all have genera that can colonise overbank alluvium as well^10^.

### The Donja Branjevina paleo-meander: animal and plant DNA

**Fig. 3:**
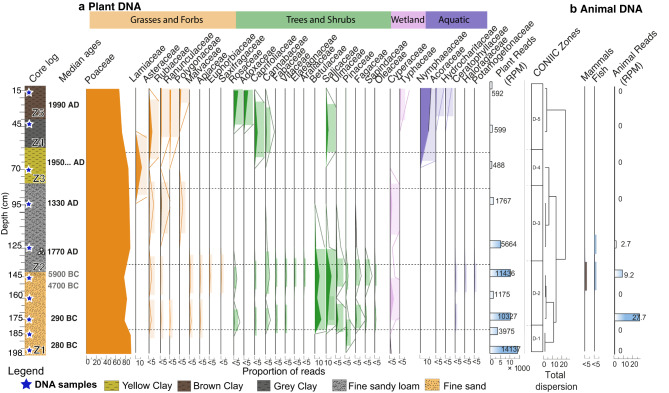
The most abundant plant and animal DNA in the Donja Branjevina paleo-meander. Composite diagram showing the proportions of ancient reads assigned to plants (**a**) and animals (**b**) at the family level (filled curve) against depth, number of reads > 500. The hollow curve indicates a 10X exaggeration. Core log is reported with sedimentation zones (see also Table S1.2 and Figure S1.1c for detailed description). The total plant and animal reads for each sample are reported as reads per million (RPM). CONIIC zone analysis is based on plant DNA detection. Colours are by category.

**Fig. 4:**
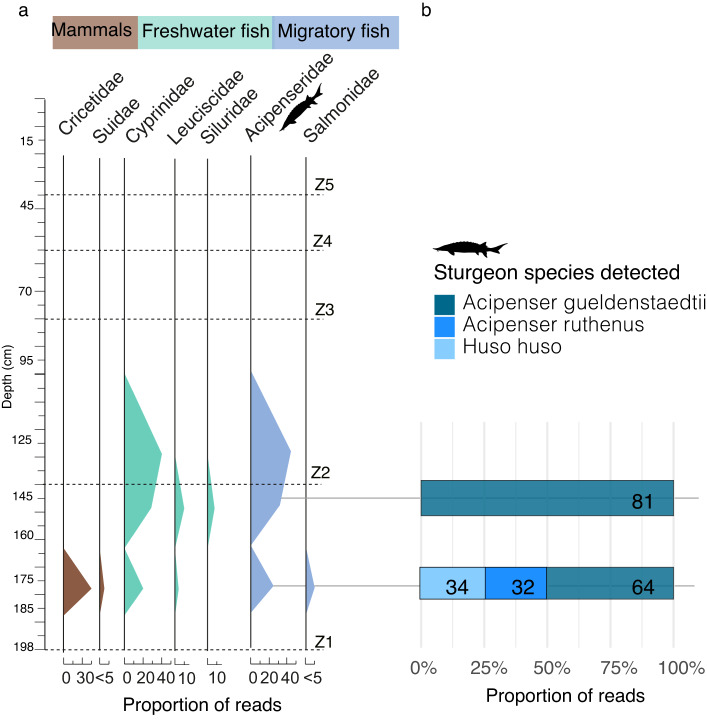
Animal DNA proportions and sturgeon species from Donja Branjevina. (**a**) Proportion of reads for animals at the family level against depth for the Donja Branjevina core. (number of reads > 100). The percentage is calculated separately from the plant reads. (**b**) Proportion of reads detected at the species level for the available sturgeon genomes.

At Donja Branjevina, we find traces of both terrestrial and freshwater ecosystems (Fig. 3); however, the radiocarbon dates and chronology seem affected by both erosional surfaces and redeposition, which limits the interpretations derived from this core. Bearing this in mind, we find that zone 1 (Z1), although dated to the Late Iron Age (~ 290−280 cal BC), includes redeposited material from the Early Neolithic (~ 5900−4700 cal BC) at a depth of 145–152 cm (Table S2.1). In this zone, we find the greatest plant diversity and detection of mammalian and fish DNA. Grasses (Poaceae) are the most prevalent plant family (60–90%) and occur together with forbs of the Asteraceae and Malvaceae, along with other flowering plants, suggesting open or at least semi-open habitats. Trees and shrubs are the second most dominant vegetation type (10%). These, based on regional pollen data^74,75^, include most likely birch (*Betula*), hazel (*Corylus*), alder (*Alnus*), hornbeam (*Carpinus*), and hophornbeam (*Ostrya*) in the family Betulaceae, along with willow (*Salix*) and poplar (*Populus)* in the Salicaceae. Sapindaceae is most likely interpreted as maple (*Acer*), which is commonly present in the region alongside the other woody taxa as part of the lowland Balkanic deciduous forest^9^. Reads from elms (Ulmaceae) and beech-oak-chestnut (Fagaceae) are less abundant, while Oleaceae sequences most likely represent ash (*Fraxinus*), although we cannot totally exclude the presence of olives (*Olea*)^75^. We also find DNA from the pine family (Pinaceae), which could be indicative of submontane forest trees, such as pines and spruce, albeit it is less common. According to^9^, the presence of pines in a lowland may be related to human intervention or the existence of degraded, dry soils, but can also be present at sandy soil pockets. Several of the identified shrubs and trees are also known to include species with edible fruits and berries, such as birch (Betulaceae), elderberry (*Sambucus*, Adoxaceae) and the cranberry bush (*Viburnum sp.*, Adoxaceae), roses/plums (Rosaceae), common lime, *Tilia*, and mallow, *Malva* (Malvaceae) and Vitaceae, a family that includes grapevines. Mammals are also detected in this sediment layer, in particular the presence of Suidae (pigs or wild boar), even if in low abundance (< 5%), which could be an indication of household livestock (Fig. 4, a).

Sedges (Cyperaceae) and aquatic plants such as pondweed, watermilfoil, and hornworts (Ceratophyllaceae, Potamogetonaceae, and Haloragaceae), are also found in this depositional zone, although in low abundance (< 5%), their presence is indicative of a wetland environment. In correspondence with the detection of aquatic plants, we also observe different fish taxa (Fig. 4). These are dominated by freshwater taxa, with carps (Cyprinidae and Leuciscidae) being the most prevalent, followed by catfish (Siluridae). Among migratory species, sturgeons (Acipenseridae) are the most abundant, with salmon (Salmonidae) also present. Considering the historical significance of sturgeon exploitation in the Danube region and the extirpation of remaining populations in the Carpathian basin, we undertook a detailed taxonomic analysis of the Acipenseridae reads (Fig. 4b). This involved alignment against available genomes of the Russian sturgeon (*Acipenser gueldenstaedtii*), beluga (*Huso huso*), and sterlet (*Acipenser ruthenus*). All three species were detected at a depth of 175 cm, with the Russian sturgeon also identified at 145 cm. It is important to note that this analysis is constrained by the availability of reference genomes within the Acipenseridae family (see Discussion for further details).

In the upper stratigraphic zone (Z2), corresponding to the period ~ 1330–1770 AD, sturgeons and carps remain detectable, but no other vertebrate DNA was identified. Importantly, in this zone, we also observe a notable decrease in the proportion of deciduous forest that disappears completely at a depth of 95 cm. Here, we only detect a high proportion of Poaceae (80%), some herbaceous plants, and sedges (Cyperaceae). The upper layers (Zones 2–5) are also associated with recent dates (~ 1950. AD) and are composed of clays. Here, trees, shrubs, and aquatic plants (Nymphaeaceae) occur alongside the dominant grasses (Poaceae), suggesting an open grassland near the riverbank with scattered woody taxa. This pattern is consistent with the presence of a canal and cultivated fields at the sampling site.

To investigate the temporal variation in plant community composition and environmental conditions, we applied CONIIC, an agglomerative stratigraphically constrained cluster method to the plant read matrix (Fig. 3, “CONIIC Zones”). The clustering method generates a dendrogram, showing the degree of dispersion between samples and aggregates, reflecting changes in the plant assemblages through the sequence. We find that zone D-5 exhibits the greatest dissimilarity compared to the other zones, followed by the division between clusters D-4/D-3 and D-2/D-1. Within the latter, we observe greater dispersion likely due to low taxonomic diversity in the basal zone (D-1), which is predominantly grass-dominated.

### The Magareći Mlin paleo-meander: animal and plant DNA

**Fig. 5:**
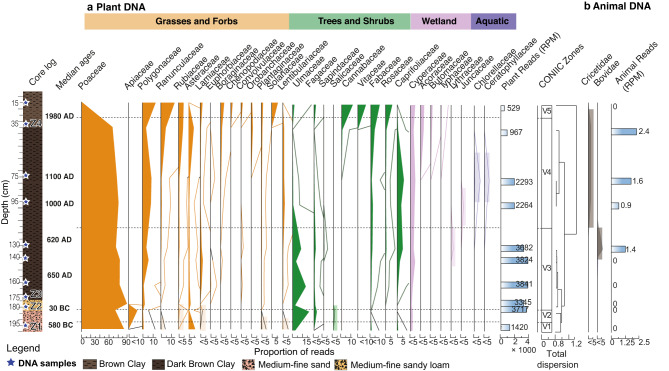
The most abundant plant and animal DNA at Magareći Mlin. Composite diagram showing the proportions of ancient reads assigned to plants (**a**) and animals (**b**) at the family level (filled curve) against depth, number of reads > 500. The hollow curve indicates a 10X exaggeration. Core log is reported with sedimentation zones (see also Table S1.2 and Figure S1.1d for detailed description). The total plant and animal reads for each sample are reported as reads per million (RPM). CONIIC zone analysis is based on plant DNA detection. Colours are by category.

At Magareći Mlin, both terrestrial and aquatic plant taxa are represented in the *seda*DNA record (Fig. 5). During the Iron Ages (~ 580−30 BC), which is the bottom layers of the core (Z1-Z2), we find a landscape predominantly composed of grasses (80%) and trees (20%), with grasses (Poaceae) and elm (Ulmaceae) being the most abundant. The presence of sedges (Cyperaceae) also in these layers represents a vegetation signal from the riverbanks. The majority of the sediment layers in this core consist of a dark brown clay layer, which also yielded the highest diversity of animal and plant DNA reads (Z3). We identify two distinct zones within this layer using the cluster analysis (Fig. 5, CONIIC zones V3-V4), which reflect a vegetation turnover during this period. In the Early Medieval period (~ 650−620 AD), grasses (Poaceae) remain the most abundant (~ 70–90%) together with elm (Ulmaceae), thus representing a continuity in the vegetation composition from the previous zones. In this section, we also observe the first occurrence of mammals within the family Bovidae, which is approximately at the same depth as the recovered mammal bones (Figure S1.2). Transitioning to the High Medieval period (~ 1000–1100 AD), the *seda*DNA record suggests an increase in human-induced alterations of the landscape. Although grasses (Poaceae) remain dominant (~ 70%), elm trees disappear, and herbaceous plants, particularly buckwheat (Polygonaceae) and the family Asteraceae, increase in abundance. These genera, often associated with ruderal and weedy species, might signal the presence of agricultural or disturbed soils^9^. Sedges (Cyperaceae) and aquatic plants (Chlorellaceae, Ceratophyllaceae) may suggest a marshy environment characteristic of the riverbanks.

The uppermost layer (Z4), dated to the more recent period (~ 1980 AD), is found to be the least similar (V-5) compared to the other zones in the cluster analysis (CONIIC). Here we detect elm (Ulmaceae) and likely oak or beech (Fagaceae) in low abundance (< 5%), while fruit-bearing shrubs, such as roses (Rosaceae), grapes (Vitaceae), and legumes (Fabaceae), are prevalent. Poaceae are less abundant, while Polygonaceae, Plantaginaceae, and buttercups (Ranunculaceae) show an increase. This pattern reflects a mosaic landscape of open and disturbed habitats, along with woody plants and shrubs that provide edible resources. These changes may be associated with shifts in land use and afforestation documented in the region^9^, consistent with post-1850s agricultural expansion and reduced burning as part of improved management practices.

## Discussion

### Environmental DNA from open-air archaeological sites: challenges and perspectives

Shotgun sequencing of environmental DNA has proven to be an efficient tool for paleo-ecological reconstruction^28,31^. However, its preservation and hence detection rely on the type of deposit and a combination of taphonomic factors, such as input biomass^76^, pH, temperature, mineral composition^77^, oxygen availability^78–80^, enzymatic and chemical degradation^81,82^, absorption and transportation^76,83–85^. For example, clay has shown to preserve extracellular DNA effectively due to its higher surface area and strong binding capacity^86^, but our results show that this trend is not consistent. For instance, layer Z2 at Donja Branjevina, composed of yellow clay exposed to oxygen and likely affected by drying (Table S1.2; Figure S1.1), exhibits lower DNA abundance than the overlying sandy layer, highlighting a still largely unknown aspect of how DNA interacts with different sediments and chemical conditions to influence its stability. These factors make it complicated to ascertain where ancient DNA can be found, its preservation state, and whether the DNA remains in situ or has migrated between layers of different ages - a process also defined as leaching^87^. In particular, the preservation of DNA from open-air archaeological sites and dried-out river/lake deposits, which are also continuously subjected to weathering, drying, freezing, and thawing cycles, might be hampered, and the context directly affected by these conditions. To mitigate potential contamination from upper layers, we established precise sediment profiles and evaluated the presence of modern DNA in older strata. Our DNA damage-age models (Figures S4.1-S4.4) enabled us to filter out low-damage reads and validate the application of data-driven thresholds.

Additionally, the fluvial environment is characterised by erosion of the riverbanks and redeposition of sediments, which likely incorporate older material into more recent deposits. In particular, these processes explain the complex chronology found in the Donja Branjevina paleo-meander, resulting in outlier dates within the sandy layers. Such outliers are indicative of an active channel and are most likely the result of fluvial erosion and sediment reworking. We suggest that the variation we observe in the proportions of terrestrial and aquatic *seda*DNA found within the sediment sections (Z1 at Donja Branjevina and Z1-Z2 at Magareći Mlin) may be influenced by localised riverbank erosion and the potential redeposition of molecules transported over long distances^76,84,88–91^. Therefore, we conservatively interpret the *seda*DNA record at Donja Branjevina as primarily reflecting the local signal rather than transported or redeposited material, mainly due to taphonomic processes of *seda*DNA^76,92^. In this regard, the detection of mammalian DNA, including pigs/boars (Suidae) and rodents (Cricetidae), alongside fish DNA in the same layer (Figs. 4 and 5), suggests that the sedaDNA originates from both the active river channel and the surrounding terrestrial environment. Further insights could potentially be gained through the application of other molecular techniques, such as sterols or other biomolecules^93^. In contrast, the stratigraphy at Magareć Mlin is mostly composed of clay, indicative of lower-energy depositional environments such as an oxbow lake and exhibits a more continuous chronology. This, together with the finding of a mammal bone fragment (Figure S1.2) and Bovidae DNA in the mid-section of Z3, suggests that the terrestrial *seda*DNA primarily reflects local sources. The relatively low proportions of animal versus plant DNA in our metagenomic datasets likely reflect the actual biomass in the landscape and do not fully represent the overall biodiversity. These challenges highlight the need for further investigation in such depositional contexts to better understand the dynamics and taphonomy of *seda*DNA.

Furthermore, the taxa identified are also affected by how well-represented each given species is in the reference database for the specific study area. A limitation that in our analysis can lead to false positives^65^. While we addressed this limitation by competitively mapping the sequences against high-quality genomes and interpreting the data at the family level, it will still miss the presence of absent genomic references. In more detail, in particular for the study at hand, only three of the five Danube sturgeon species historically inhabiting this region, have fully sequenced genomes (sterlet, Russian, and beluga sturgeon), which means that potential detection of the ship sturgeon (*A. nudiventris*) and stellate sturgeon (*A. stellatus*) from *seda*DNA would rely on the limited publicly available short mitochondrial and nuclear markers. We assume that sequencing the complete genomes of these under-represented taxa would improve the detection of sturgeon species identification in metagenomic datasets. Similarly, expanding genomic references to include more plant genera native to the region would strengthen *seda*DNA-based reconstructions of past vegetation.

### Human impacts and vegetation dynamics

At Donja Branjevina and Vinča-Belo brdo, we identify a variety of plants showing fluctuations in woodland and open-steppe vegetation. Our results indicate that *seda*DNA reflects the local plant composition, as evidenced by the consistent recovery of wild pear (*Pyrus* sp., Rosaceae), elderberry (*Sambucus*, Adoxaceae), apple (*Malus* sp., Rosaceae), and grape (*Vitis vinifera*, Vitaceae), all of which are reported in pollen and macrofossil records from Vinča-Belo brdo and from the Mesolithic contexts of Vlasac^72,94^ and Lepenski Vir^95^ on the Danube Gorges as well as the Neolithic sites of Divostin, Grivac, Opovo and Starčevo in central Serbia (see Results)^10,70,96^.

Donja Branjevina and Magareći Mlin exhibit similar ecosystem dynamics, marked by a decline in woodland coverage in the middle portion of the sequences. In particular, the proportion of elms (Ulmaceae) seems to significantly reduce starting from the period ~ 1000–1100 cal AD at Magareći Mlin and between ~ 1330–1770 cal AD at Donja Branjevina. However, these changes may also include alterations in hydrology, soil composition, or land management practices, and it could be linked with the sporadic evidence of medieval occupancy in the area^25,97–99^, but these conclusions should be taken with caution for the site of Donja Branjevina, since they might also result from the redeposition and erosion processes affecting this zone (Z2). At the moment, there is no pollen data for the period covered by our cores, and this lack of independent paleoecological records limits direct comparisons. Our *seda*DNA results, therefore, provide a first line of evidence for vegetation dynamics in the region during this interval.

Particularly noteworthy is the detection of DNA from three sturgeon species, which are now critically endangered in the Danube basin and extirpated in the study region. The main cause of disruption of their migratory routes from the Black Sea is primarily due to the construction of large hydropower plants in the Danube Gorges during the 20th century, specifically Djerdap I (1972) and Djerdap II (1984) on the Serbia-Romania border, together with overfishing and habitat pollution^100,101^. Although sturgeon remains are absent from the Late Iron Age period (~ 280 BC) at Donja Branjevina, both archaeological evidence (see Fig. 1a for fish assemblages) and historical records indicate intensive sturgeon fishing in the Danube within the Bačka region, where Donja Branjevina and Magareći Mlin are located. During this period, sturgeon migratory routes extended as far upstream as the Austrian and Bavarian sections of the river^15,102,103^. This is in line with the genetic findings of *H. huso* and *A. gueldenstaedtii* identified in the *seda*DNA and further supports the historical presence of these species in the region.

Ongoing archaeological work might help to integrate and support our *seda*DNA identifications. Furthermore, recent advancements in target-enrichment hybridization capture applied to ancient sediments, as demonstrated by Slon et al. (2017)^104^, Kjær et al. (2022)^31^ and others^105–108^, might have the potential to improve the detection of animal DNA also in an open-air sites. For example, the use of fish-specific baits^109–111^, could significantly improve the ability to track ancient sturgeon DNA in historic river sediments.

## Conclusion

The complexity of sedimentary ancient DNA (*seda*DNA) from Neolithic archaeological deposits and paleo river channels along the Danube in the Carpathian Basin may not be completely understood. In this study, we show that *seda*DNA can reveal changes in both freshwater and terrestrial ecosystems dating back thousands of years, and these findings align with other paleobotanical proxies and faunal evidence. Importantly, we can recover ancient DNA even when other archaeological organic materials are not well preserved. However, our results highlight a methodological challenge in *seda*DNA research. Low-energy, stable depositional environments provide more reliable chronologies but are often dominated by terrestrial DNA, limiting the recovery of aquatic taxa such as fish. In contrast, active river channels may offer stronger aquatic signals but complicate chronological interpretation due to erosion and sediment redeposition. In many landscapes, paleo-meanders are the only feasible option for investigating aquatic signals in proximity to an archaeological context, due to the spatial and temporal variability of rivers. Addressing this challenge requires an integrated approach, combining high-resolution sediment composition analyses and radiocarbon dating, as well as geoarchaeological techniques such as sediment micromorphology for the identification of fine-scale stratigraphic structures. In addition, molecular strategies such as targeted enrichment of fish DNA can help overcome terrestrial inputs. More research is necessary to better understand how water movement affects the breakdown and redeposition of ancient DNA in river environments and the processes influencing open-air archaeological deposits. Although using *seda*DNA in archaeological studies is still in development, combining it with traditional archaeological techniques holds great promise for tracking human-induced landscape changes. This is particularly relevant given that most archaeological sites across the globe are found in areas where ancient DNA embedded in layers is exposed to fluctuations under unprotected environmental conditions. Consequently, unlocking the paleo-genetic record is essential for a more comprehensive understanding of the human past. Lastly, our findings show that *seda*DNA can effectively track past distributions of species such as sturgeons, which are currently threatened and, in some areas, extirpated due to human exploitation and landscape management practices.

## Supplementary Information

Below is the link to the electronic supplementary material.

Supplementary Material 1

Supplementary Material 2

## Data Availability

The sequencing data analysed in this article (fastq files) and the associated sample metadata have been deposited in the European Nucleotide Archive (ENA) under the project accession number PRJEB81156. The codes, raw data and Rscripts are available on Zenodo at the permanent DOI 10.5281/zenodo.17546741.
