# Supplementary material for: Ancient DNA reconstruction of Late Holocene ecosystems within the Carpathian Basin from paleo-meanders and archaeological deposits
Source: Sci Rep. 2026 Feb 3;16:4301. doi: 10.1038/s41598-026-35509-2 (PMC12868805; doi:10.1038/s41598-026-35509-2)
Supplement: Supplementary file 2 — Supplementary Material 2 [file 41598_2026_35509_MOESM2_ESM.docx]

Ancient DNA reconstruction of Late Holocene ecosystems within the Carpathian Basin from paleo-meanders and archaeological deposits

Giulia Zampirolo^1^, Anthony H. Ruter^2^, Ivana Živaljević^3^, Kristina Penezić^4^, Philip Francis Thomsen^5^, David Orton^6^, Milica Kašanin Grubin^7^, Nevena Antić^7^, Mikkel Winther Pedersen^2^

^1^Section for Molecular Ecology and Evolution, Faculty of Health and Medical Sciences,

Globe Institute, University of Copenhagen, Copenhagen, Denmark.

^2^Centre for Ancient Environmental Genomics, Faculty of Health and Medical Sciences, Globe Institute, University of Copenhagen, Copenhagen, Denmark

^3^ Department of History, Faculty of Philosophy, University of Novi Sad, Serbia.

^4^ BioSense Institute, University of Novi Sad, Novi Sad, Serbia.

^5^ Department of Biology, Aarhus University, Denmark.

^6^ BioArCh, Department of Archaeology, University of York, UK.

^7^ Institute of Chemistry, Technology and Metallurgy, University of Belgrade, Serbia.

**Supplemental Material**

# 1. Sediment composition of the paleo-meander deposits


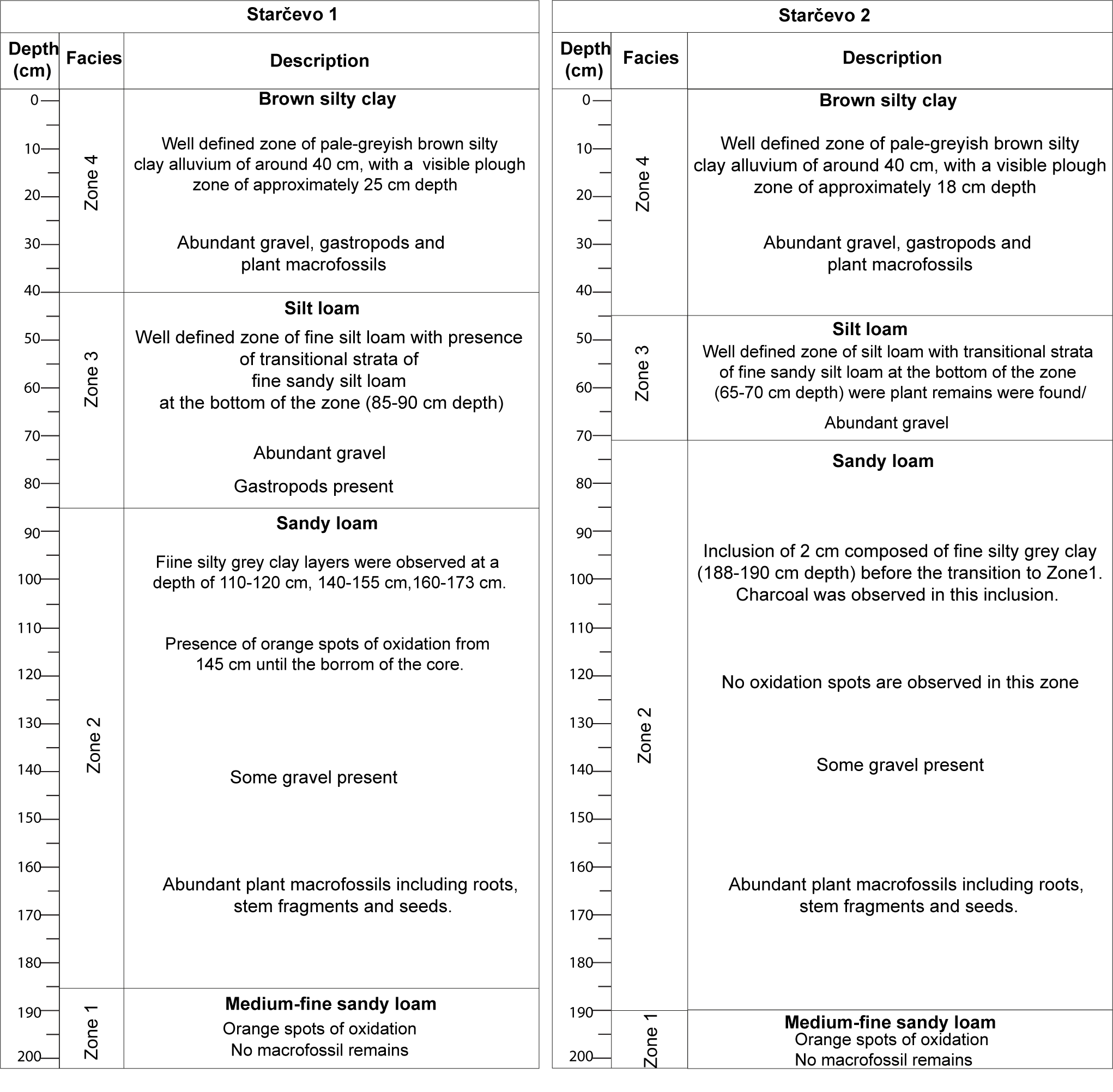


**Table S1.1.** **Description of the stratigraphy and macrofossil record of the Starčevo-Grad cores.**


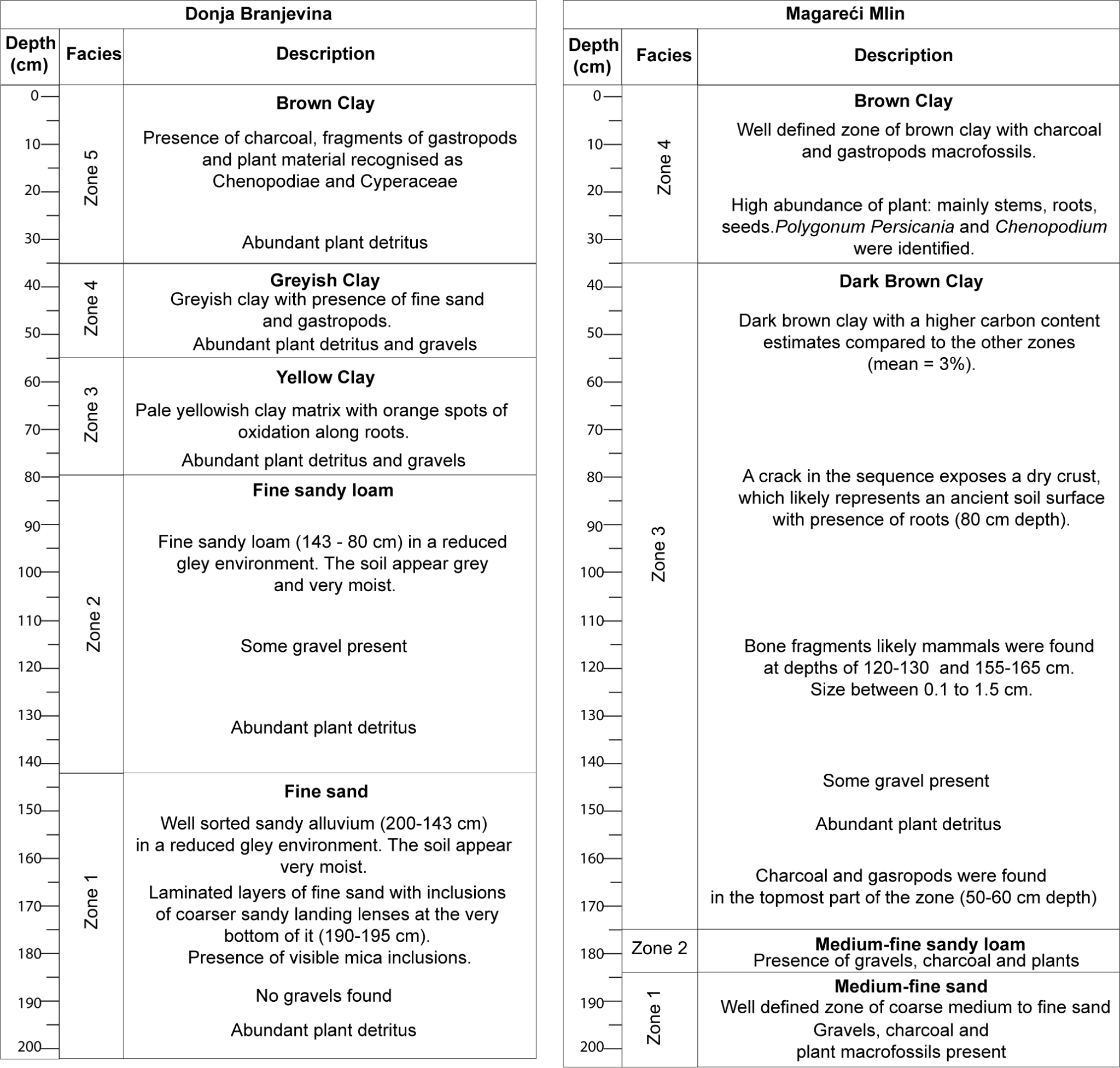


**Table S1.2.** **Description of the stratigraphy and macrofossil record of the Donja Branjevina and Magareći Mlin cores.**


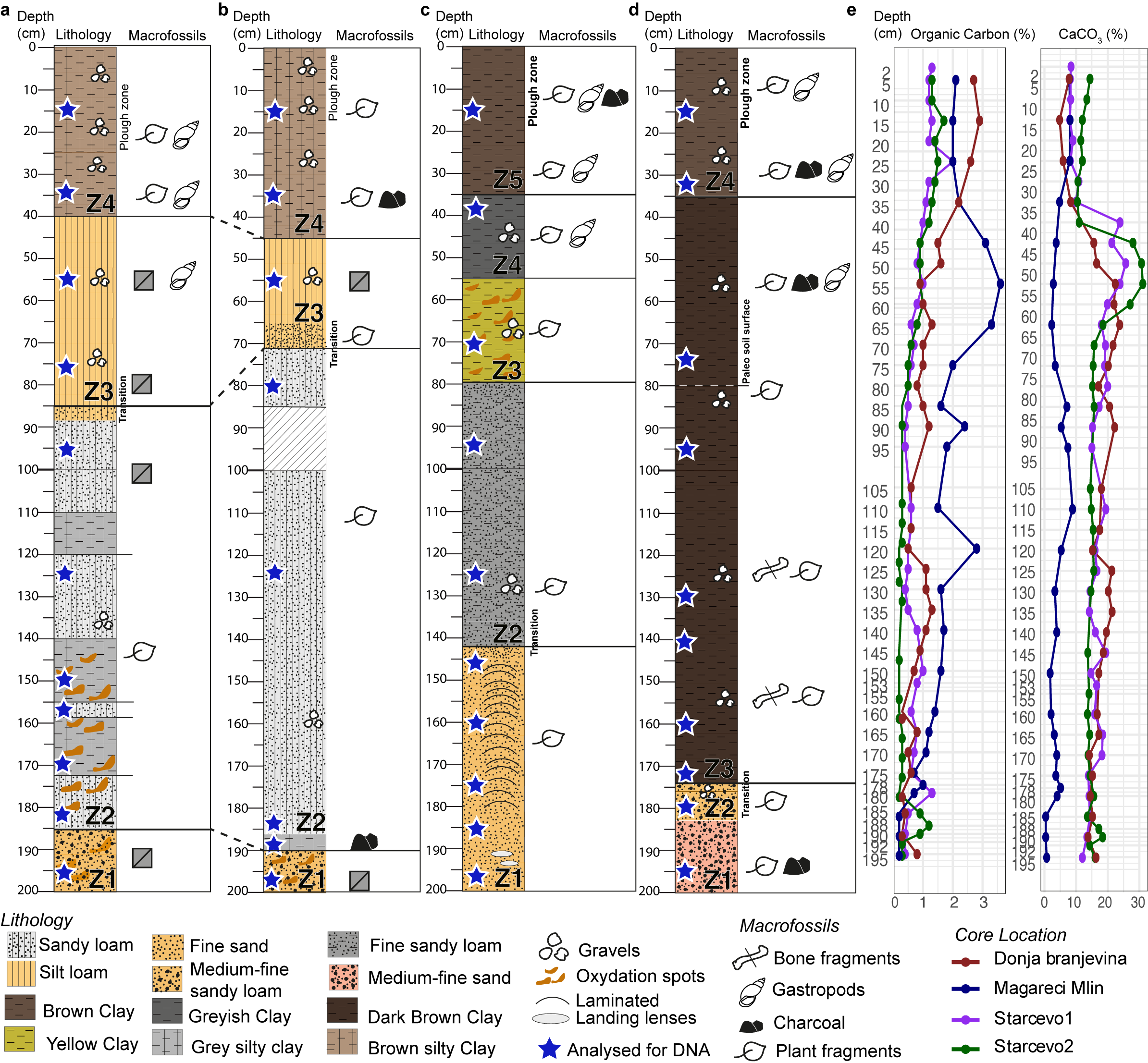


**Figure S1.1. Comparative analysis of the cores’ sedimentology and organic matter.** Sedimentary deposition layers with macrofossil identification for STR 1 (**a**), STR 2 (**b**), DOB2 (**c**), MML1 (**d**) cores; Organic Carbon (OC) and Calcium Carbonate (CaCO_3_) concentrations (**e**).

**Figure S1.2.** **Fragment of bone found at a depth of 135 cm in the Magareći Mlin core.**
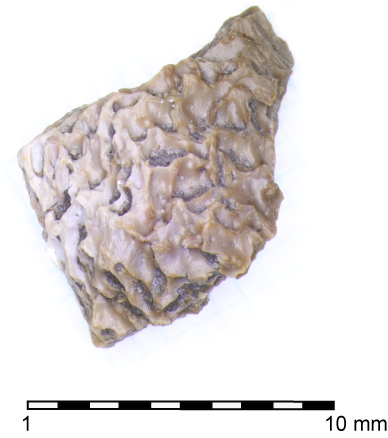


# 2. Chronological data and background information

| **Laboratory Code** | **Depth (cm)** | **Site** | **Core** | **Material** | **Date (14C years BP)** | **Calibrated age 95.4% probability (AD)** | **Calibrated age 95.4% probability (BC)** |
| --- | --- | --- | --- | --- | --- | --- | --- |
| UCIAMS-286524 | 25-28 | Magareći Mlin | 1.1 | plant detritus | - | 1953-2015* | - |
| UCIAMS-286525 | 75-78 | Magareći Mlin | 1.1 | plant detritus | 935±15 | 1040-1159 | - |
| UCIAMS-286526 | 95-98 | Magareći Mlin | 1.1 | plant detritus | 1025±15 | 993-1030 | - |
| UCIAMS-286527 | 125-126 | Magareći Mlin | 1.2 | plant detritus | 1435±15 | 600-650 | - |
| UCIAMS-286528 | 155-160 | Magareći Mlin | 1.2 | plant detritus | 1375±15 | 644-667 | - |
| UCIAMS-286529 | 180-182 | Magareći Mlin | 1.2 | plant detritus | 1990±15 | 106 | 41 |
| UCIAMS-286531 | 195-198 | Magareći Mlin | 1.2 | twigs | 2455±15 | - | 751-419 |
| UCIAMS-286532 | 25 | Donja Branjevina | 2.1 | plant detritus | - | 1952-2019* | - |
| UCIAMS-286533 | 45 | Donja Branjevina | 2.1 | sedges | - | 1950…* | - |
| UCIAMS-286534 | 65 | Donja Branjevina | 2.1 | plant detritus | - | 1952-2018* | - |
| UCIAMS-286535 | 95 | Donja Branjevina | 2.1 | plant detritus | 685±15 | 1278-1380 | - |
| Beta - 698549 | 133-135 | Donja Branjevina | 2.2 | sedges | 220 ± 30 | 1731 - 1806 | - |
| Beta-696818 | 150-152 | Donja Branjevina | 2.2 | sedges | 5840±30 | - | 4791 - 4611 |
| UCIAMS-286536 | 145-147 | Donja Branjevina | 2.2 | plant detritus | 7020±180 | - | 6245-5565 |
| UCIAMS-286537 | 175-177 | Donja Branjevina | 2.2 | plant detritus | 2225±15 | - | 372-203 |
| UCIAMS-286538 | 195-197 | Donja Branjevina | 2.2 | plant detritus | 2210±15 | - | 361-200 |
| UCIAMS-286521 | 32-35 | Starčevo-Grad | 1.1 | plant | - | 1950…* | - |
| UCIAMS-286541 | 110-113 | Starčevo-Grad | 1.2 | plant | 30±15 | 1705-1909 | - |
| UCIAMS-286542 | 175-190 | Starčevo-Grad | 1.2 | plant detritus | 865±15 | 1164-1220 | - |
| UCIAMS-286522 | 30 | Starčevo-Grad | 2.1 | plant detritus | - | 1953-2020* | - |
| UCIAMS-286523 | 68-72 | Starčevo-Grad | 2.1 | plant detritus | 15±15 | 1707-1908 | - |
| UCIAMS-286543 | 116-118 | Starčevo-Grad | 2.2 | sedges | - | 1951-1960* | - |

**Table S2.1.** **Chronometric data for the three sediment cores.** All dates were calibrated in Oxcal v 4.4.4 [[1]](https://paperpile.com/c/dTR38M/tRQbF) using the IntCal20 [[2]](https://paperpile.com/c/dTR38M/jhWIW). Samples with an asterisk were calibrated using the NH1 Bomb curve of INTCAL20’s calibration curve compendium [[2]](https://paperpile.com/c/dTR38M/jhWIW).

| **Location** | **Core ID** | **Coordinates** | **Distance from the active channel (Km)** | **Distance from the archaeological site (Km)** |
| --- | --- | --- | --- | --- |
| Starčevo-Grad (Banat District, Vojvodina) | STR1 | 44°48'36.90"N, 20°40'58.50'' E | 2.8 | 1.8 |
|  | STR2 | 44°48'37.00'' N, 20°40'59.90'' E | 2.8 | 1.8 |
| Donja Branjevina (Bačka District, Vojvodina) | DOB2 | 45°27'29.41"N, 19° 8'9.07"E | 11.0 | 0.15 |
| Magareći Mlin (Bačka District, Vojvodina) | MML1 | 45°37'51.40"N, 19° 1'25.00"E | 5.6 | 0.28 |

**Table S2.2. Locations of the sediment cores analysed in this study.**


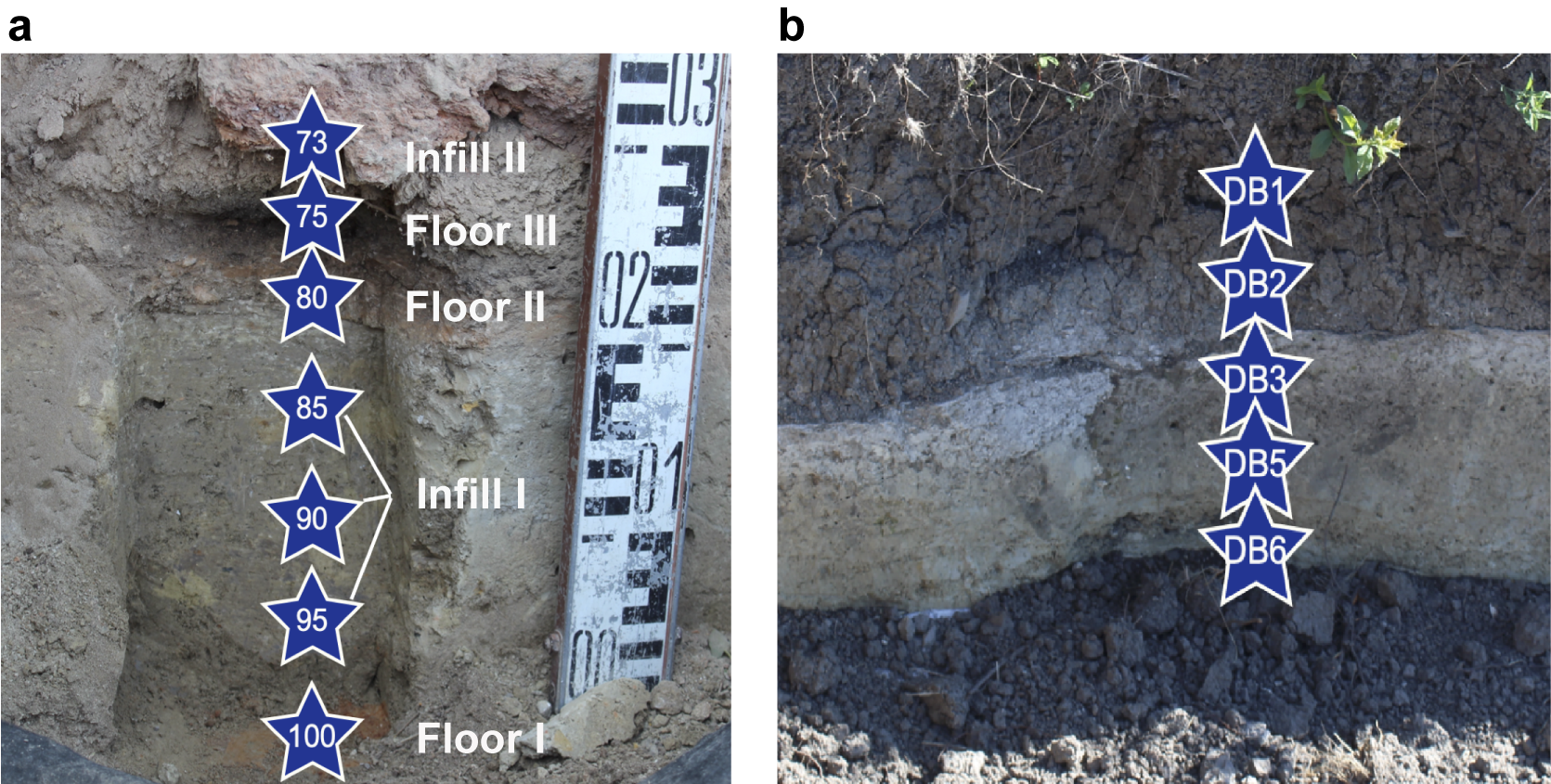


**Figure S2.1.** **Subsampling the archaeological sites of Vinča - Belo brdo (a) and Donja Branjevina (b).**

# 3. Library quality and mapping statistics


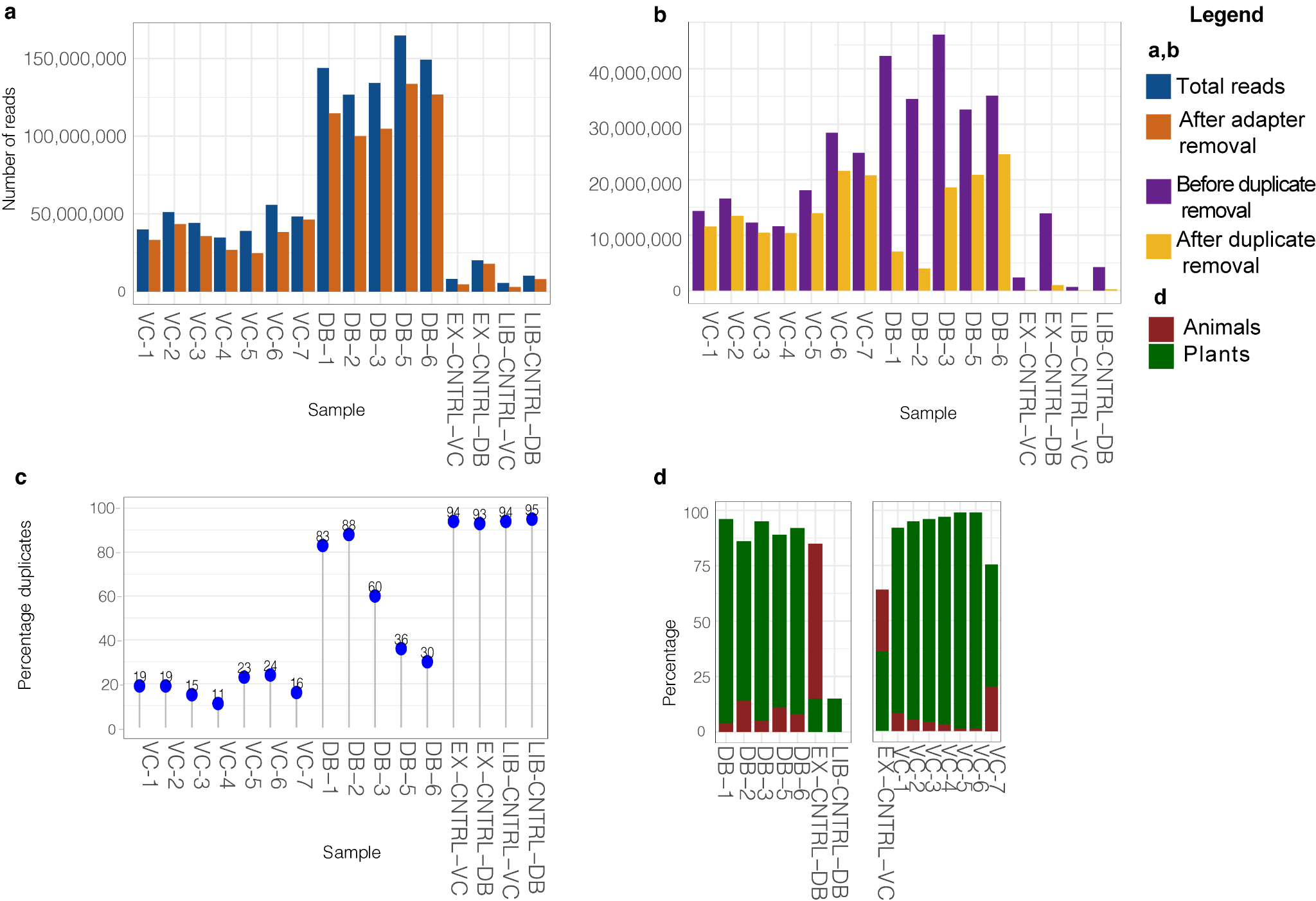


**Figure S3.1. Mapping statistics for the archaeological deposits of Donja Branjevina and Vinča-Belo brdo.**

**a.** Total reads sequenced per library and number of reads after adapter removal. **b.** Number of reads before and after duplicate removal. **c.** Percentage of duplicates removed before mapping. **d.** Proportion of reads assigned to plants and animals before applying any threshold. VC=Vinča-Belo brdo, DB= Donja Branjevina.


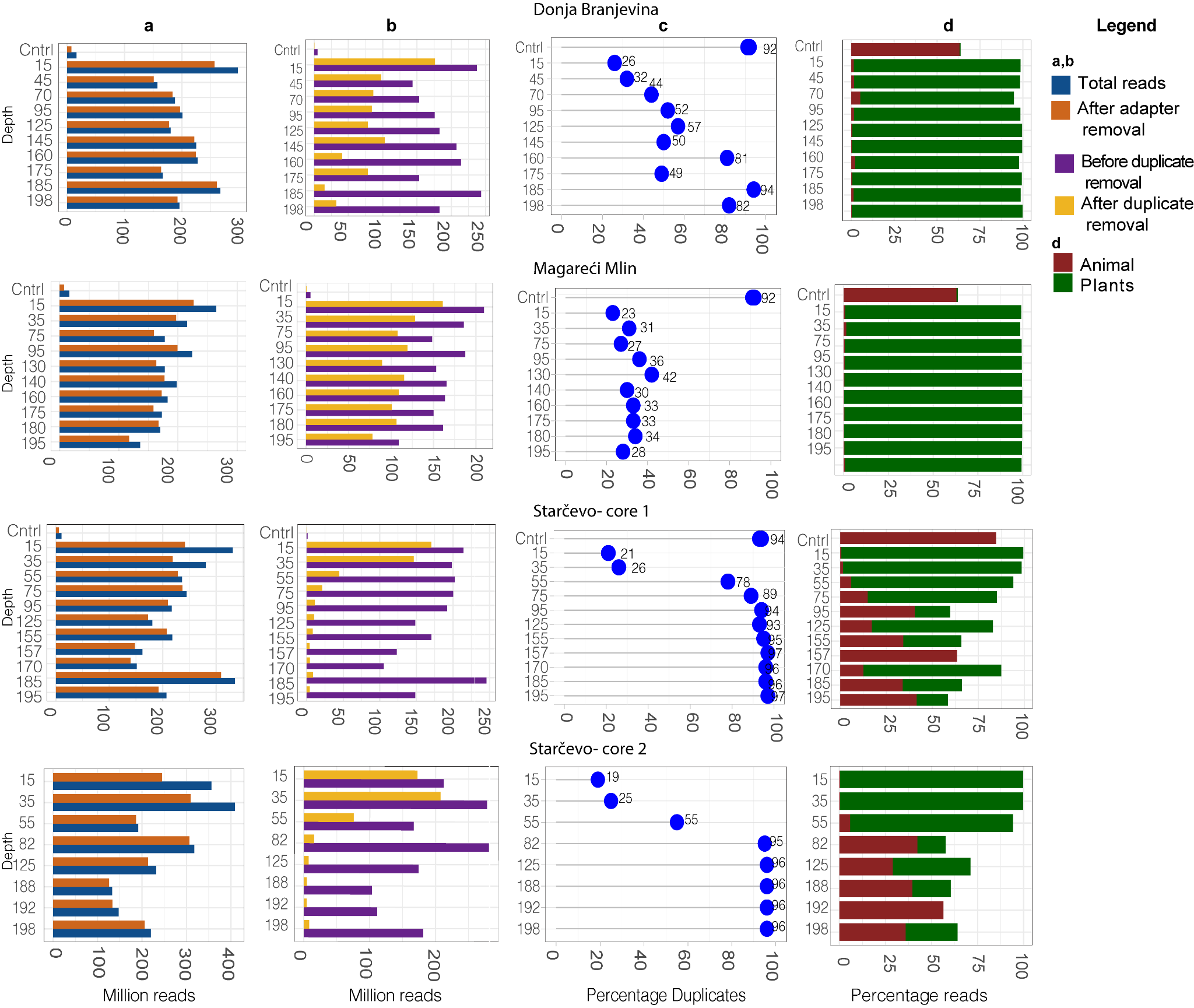


**Figure S3.2. Statistics for Donja Branjevina, Magareći Mlin, Starčevo core1 and core2.**

**a.** Total reads sequenced per library and number of reads after adapter removal. **b.** Number of reads before and after duplicate removal. **c.** Percentage of duplicates removed before mapping. **d.** Proportion of reads assigned to plants and animals before applying any threshold.

# 4. MetaDMG statistics


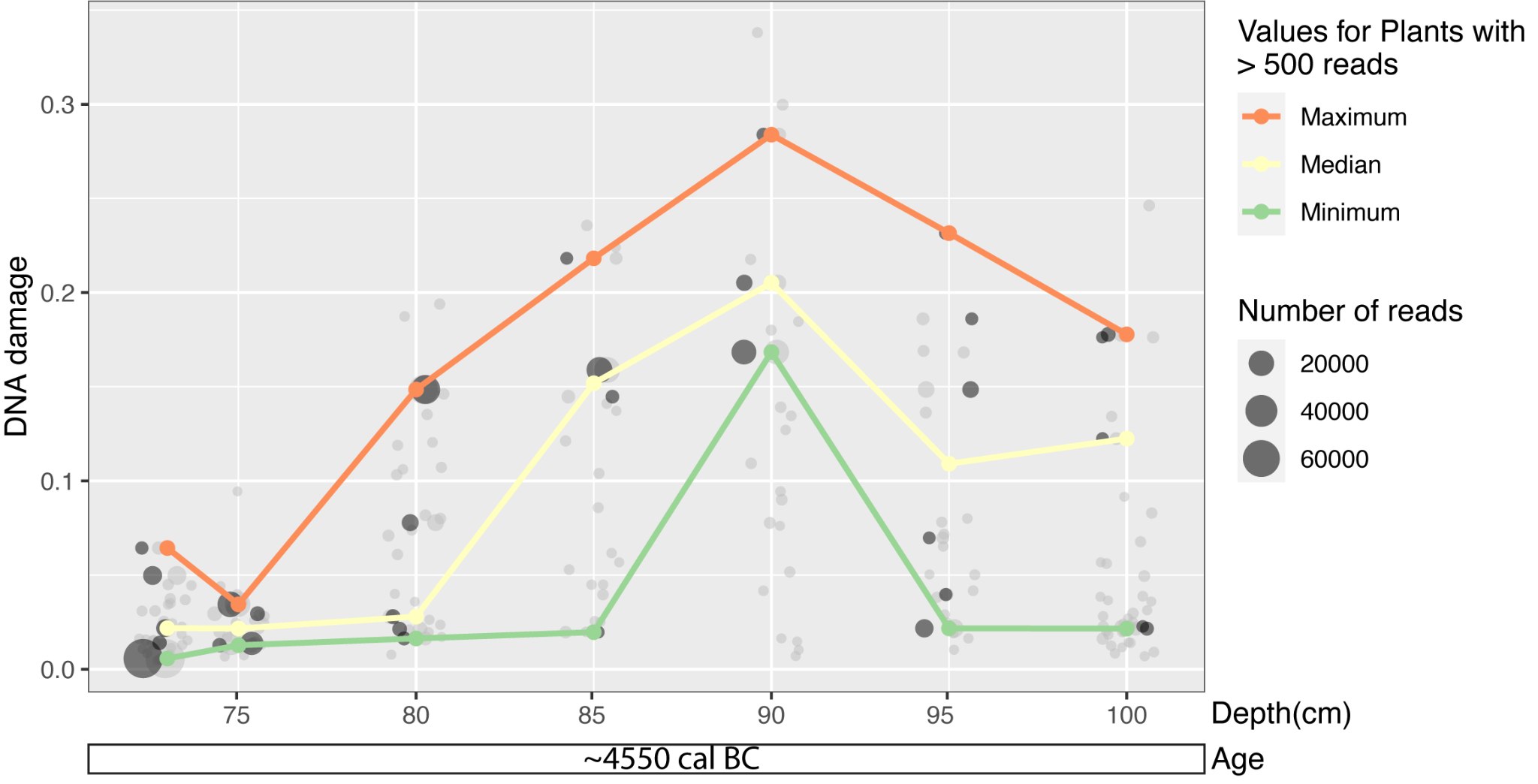


**Figure S4.1.** **Depth-damage model from Vinča-Belo brdo dataset**. Depth-damage model showing the degree of post-mortem DNA degradation of the reads assigned to terrestrial plants at the family level. The degree of damage is displayed on the y-axis, while the depth of the excavation layers is shown on the x-axis. These layers span the Late Neolithic period (~4550 cal BC). The maximum, median, and minimum values of post-mortem DNA damage at 500 read counts are highlighted in red, yellow, and green, respectively. The grey dots indicate all plant family records equal to or above 100 reads. The size of the dots corresponds to the number of reads per observation.


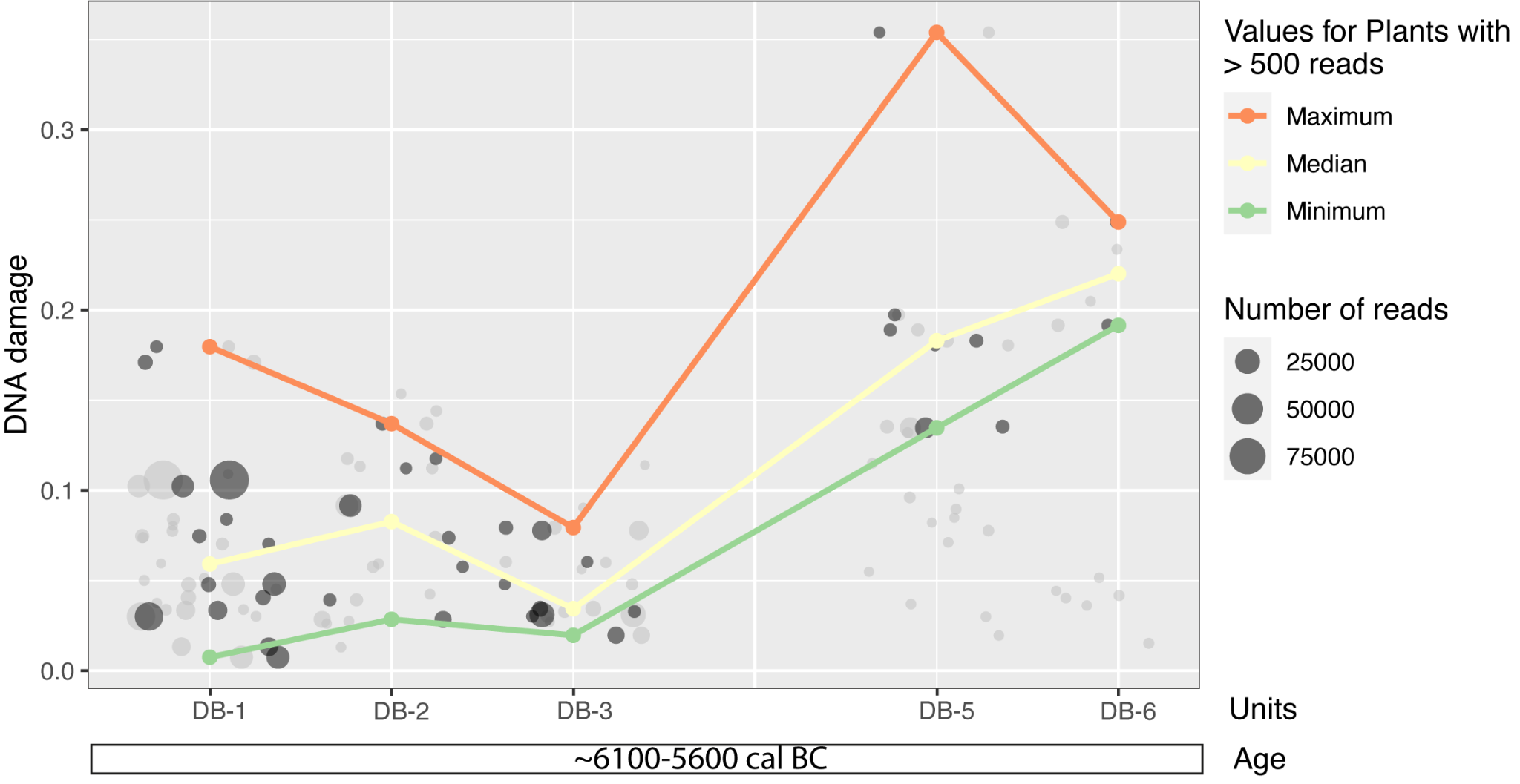


**Figure S4.2**. **Depth-damage model based on the Donja Branjevina dataset (archaeological site).** Depth-damage model showing the degree of post-mortem DNA degradation of the reads assigned to terrestrial plants at the family level. The degree of damage is displayed on the y-axis, while the stratigraphic units are shown on the x-axis. These layers span the Neolithic period (~6100-5600 cal BC). The maximum, median, and minimum values of post-mortem DNA damage at 500 read counts are highlighted in red, yellow, and green, respectively. The grey dots indicate all plant family records equal to or above 100 reads. The size of the dots corresponds to the number of reads per observation.


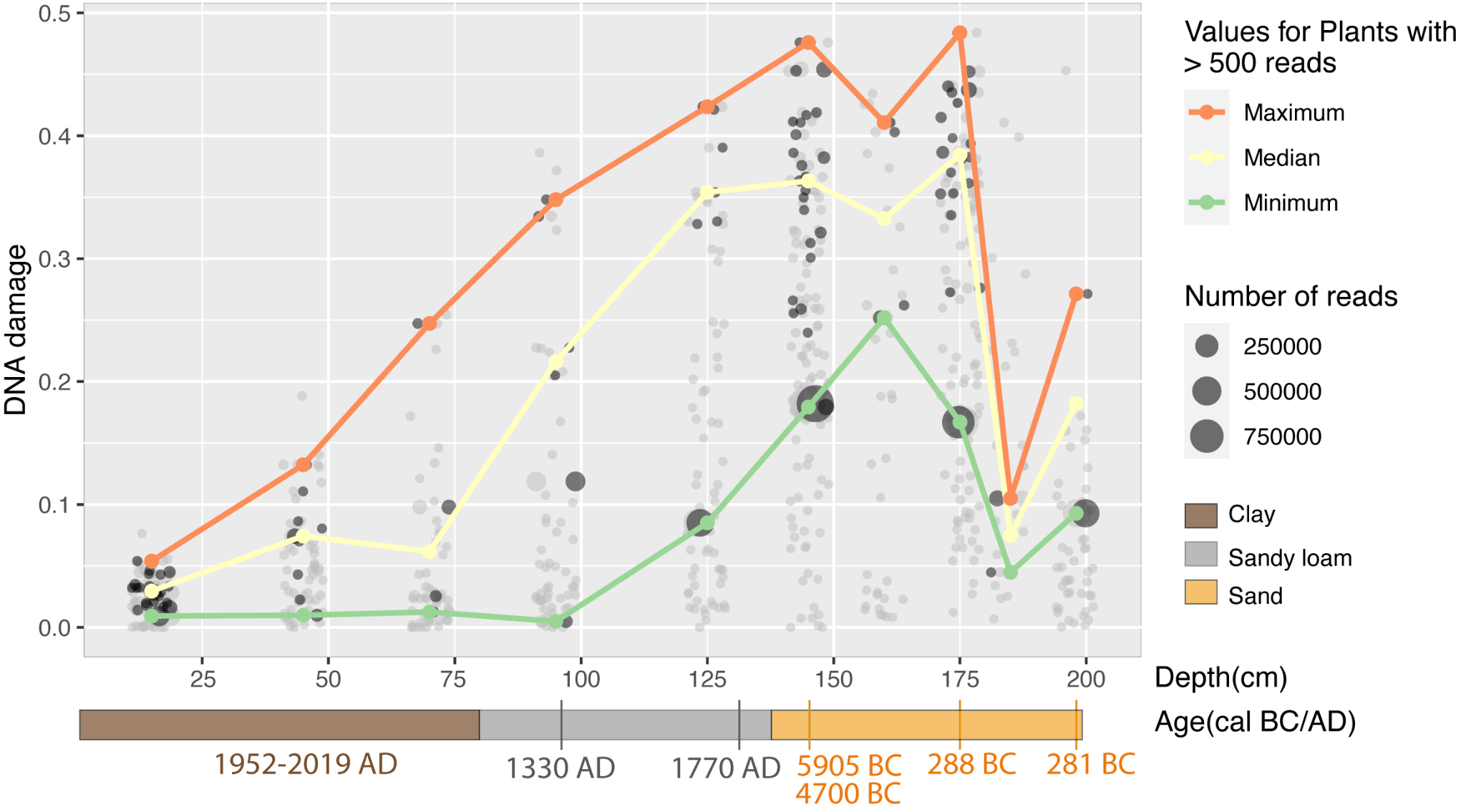


**Figure S4.3. Depth-damage model based on the Donja Branjevina core.** Depth-damage model showing the degree of post-mortem DNA degradation of the reads assigned to terrestrial plants at the family level. The degree of damage is displayed on the y-axis, while the depth and age (cal BC/AD) are shown on the x-axis. Sediment composition is also reported. The maximum, median, and minimum values of post-mortem DNA damage at 500 read counts are highlighted in red, yellow, and green, respectively. The grey dots indicate all plant family records equal to or above 100 reads. The size of the dots corresponds to the number of reads per observation.

**
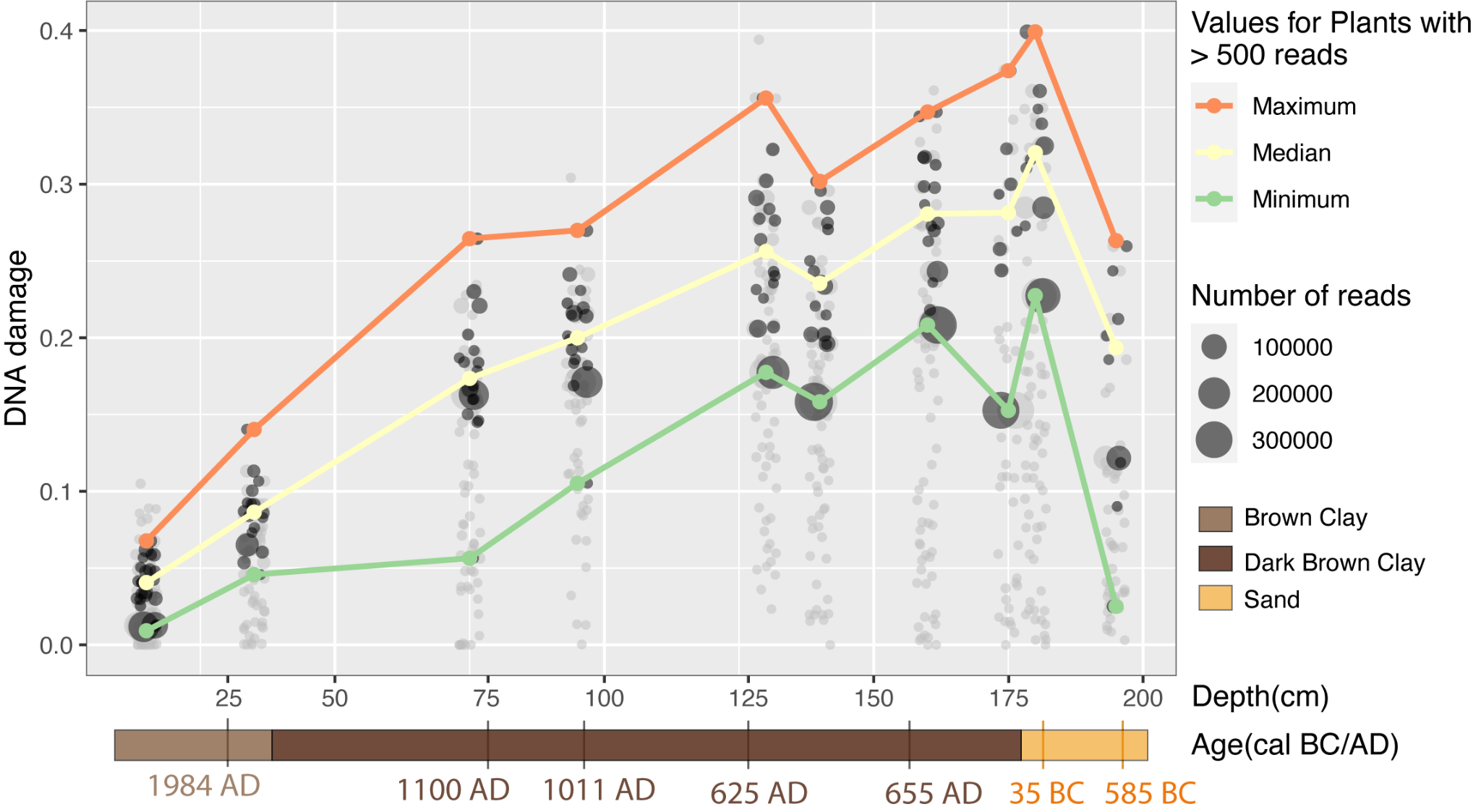
**

**Figure S4.4.** **Depth-damage model based on the Magareći mlin dataset.** Depth-damage model showing the degree of post-mortem DNA degradation of the reads assigned to terrestrial plants at the family level. The degree of damage is displayed on the y-axis, while the depth and age (cal BC/AD) are shown on the x-axis. Sediment composition is also reported. The maximum, median, and minimum values of post-mortem DNA damage at 500 read counts are highlighted in red, yellow, and green, respectively. The grey dots indicate all plant family records equal to or above 100 reads. The size of the dots corresponds to the number of reads per observation.

**
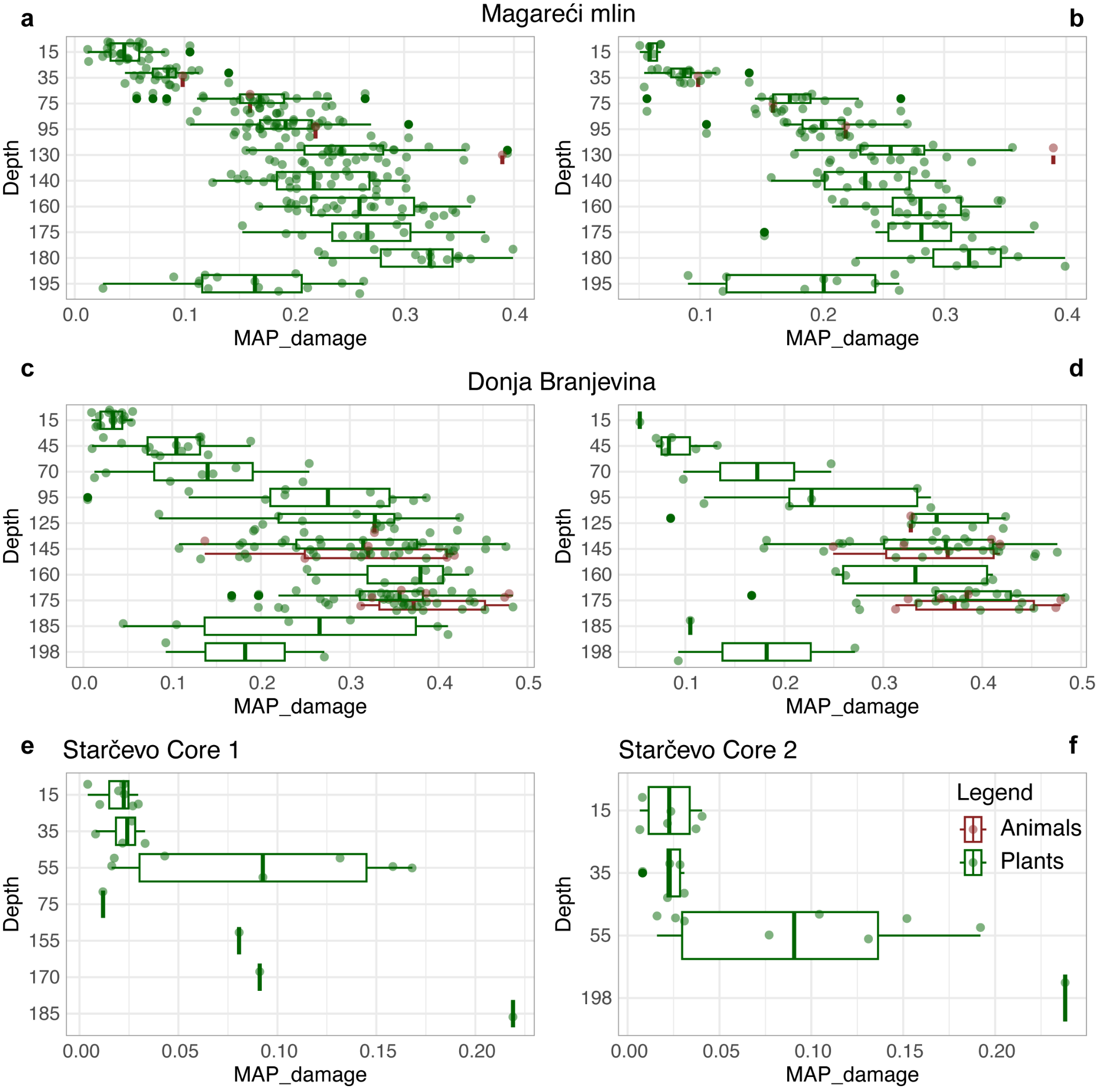
**

**Figure S4.5. Animal and plant DNA by depth for all sampling locations.**

The degree of post-mortem DNA damage is shown on the x-axis, while sample depth is displayed on the y-axis. Plant and animal reads are parsed at the family level with a significance > 2 and number of reads > 100 for Magareći mlin core (**a**), Donja Branjevina core (**c**), Starčevo core 1 (**e**) and Starčevo core 2 (**f**). The damage profiles for Magareći Mlin (**b**) and Donja Branjevina cores (**d**) are also displayed after filtering animal and plant reads according to the depth-damage model (see also figures S4.3-S4.4).


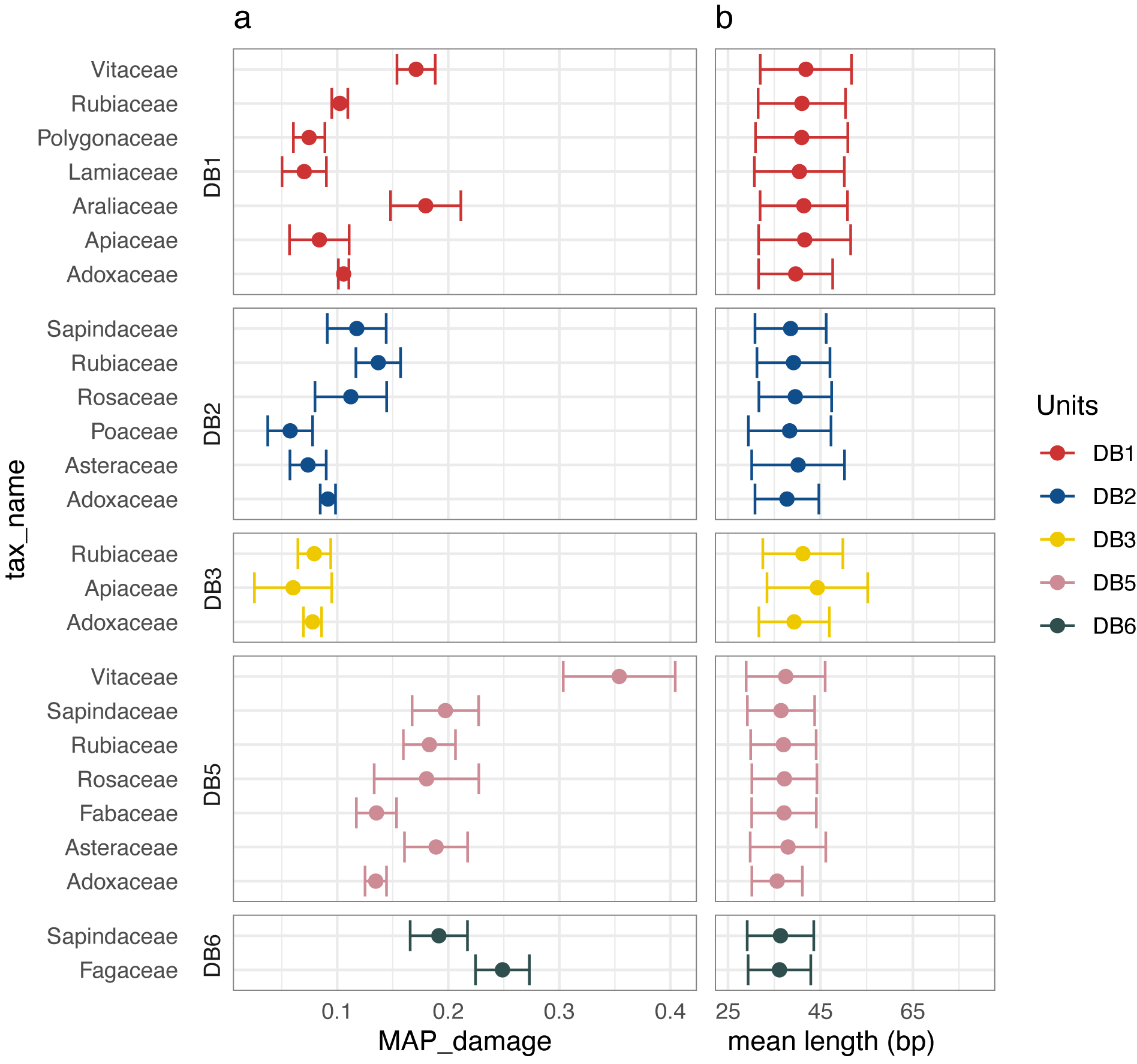


**Figure S4.6. Damage estimation (a) and mean length (b) of the most abundant plants at Donja Branjevina (archaeological deposit).** The dataset was parsed using a depth-damage model (refer to Figure S4.2) and presented at the family level. Read counts above 500.


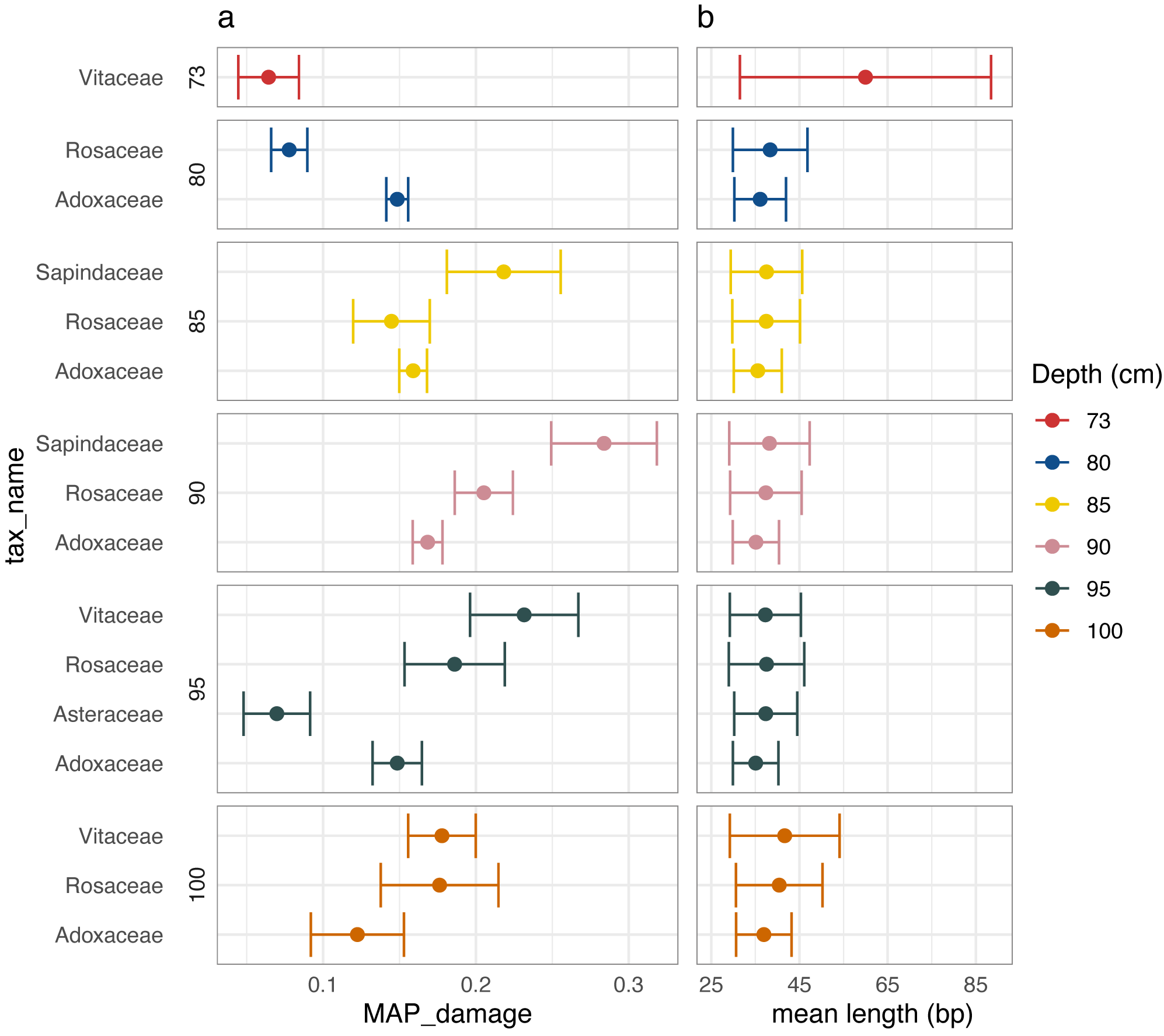


**Figure S4.7. Damage estimation (a) and mean length (b) of the most abundant plants at Vinča- Belo brdo.** The dataset was parsed using a depth-damage model (refer to Figure S4.1) and presented at the family level. Read counts above 500.

**
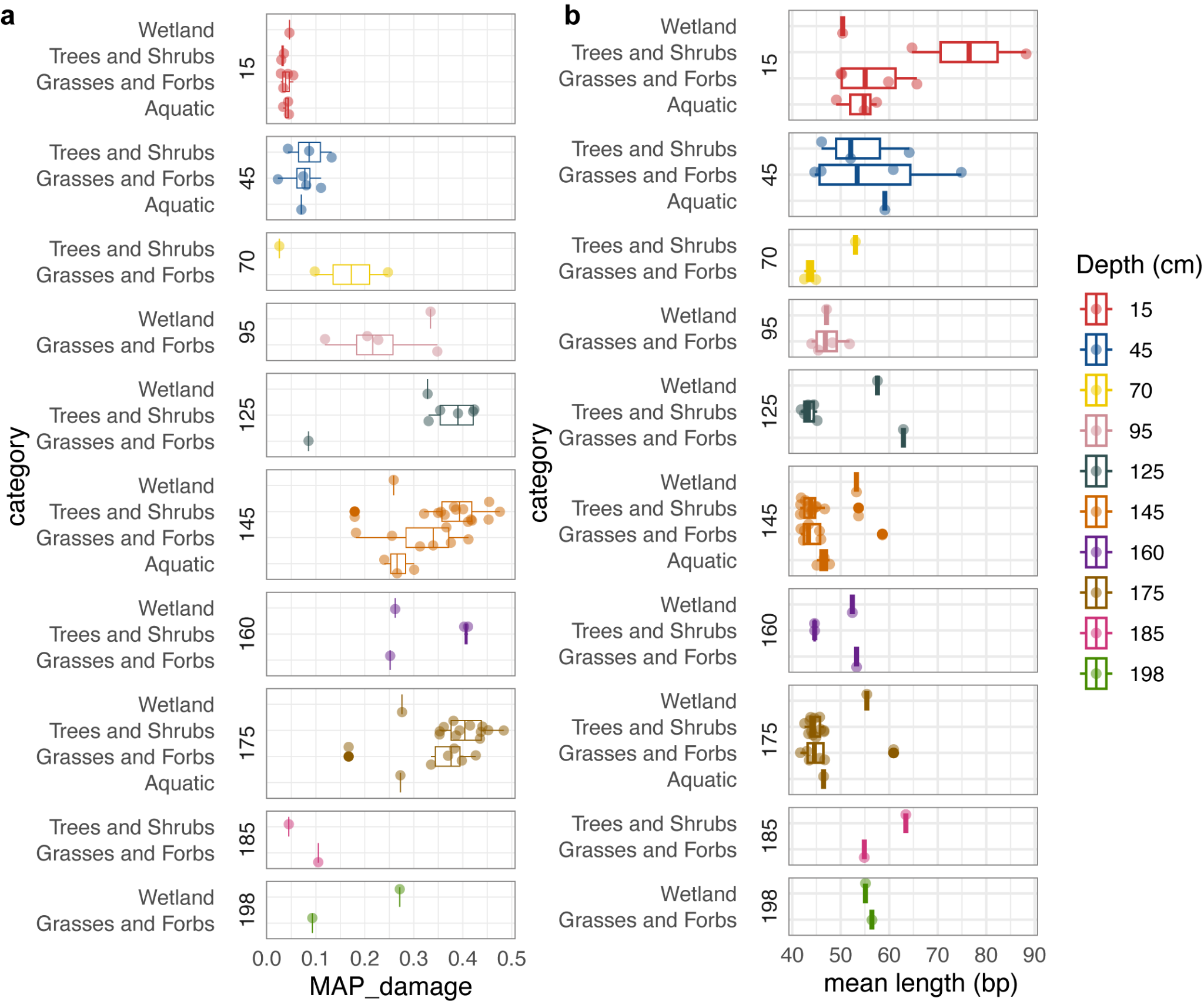
**

**Figure S4.8. Damage estimation (a) and mean length (b) of the most abundant plants at Donja Branjevina (core).** The dataset was parsed using the depth-damage model (refer to Figure S4.3). Taxonomic categories are assigned at the family level, based on read counts above 500, and are consistent with those shown in Figure 3.

**
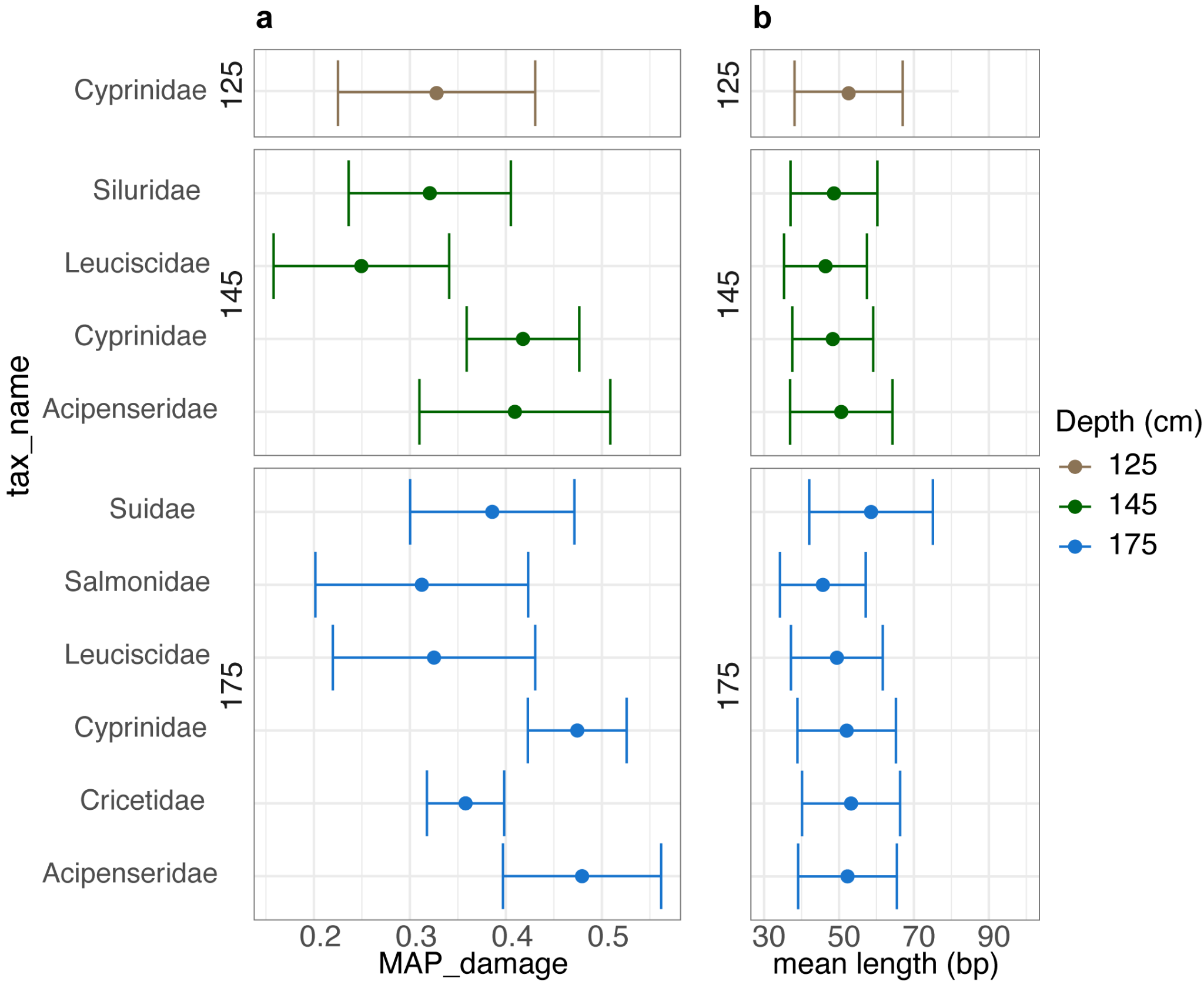
**

**Figure S4.9. Damage estimation (a) and mean length (b) of the most abundant animals at Donja Branjevina.** The dataset was parsed using the depth-damage model (refer to Figure S4.3). Taxonomic categories are assigned at the family level, based on read counts above 100, and are consistent with those shown in Figure 3.

**
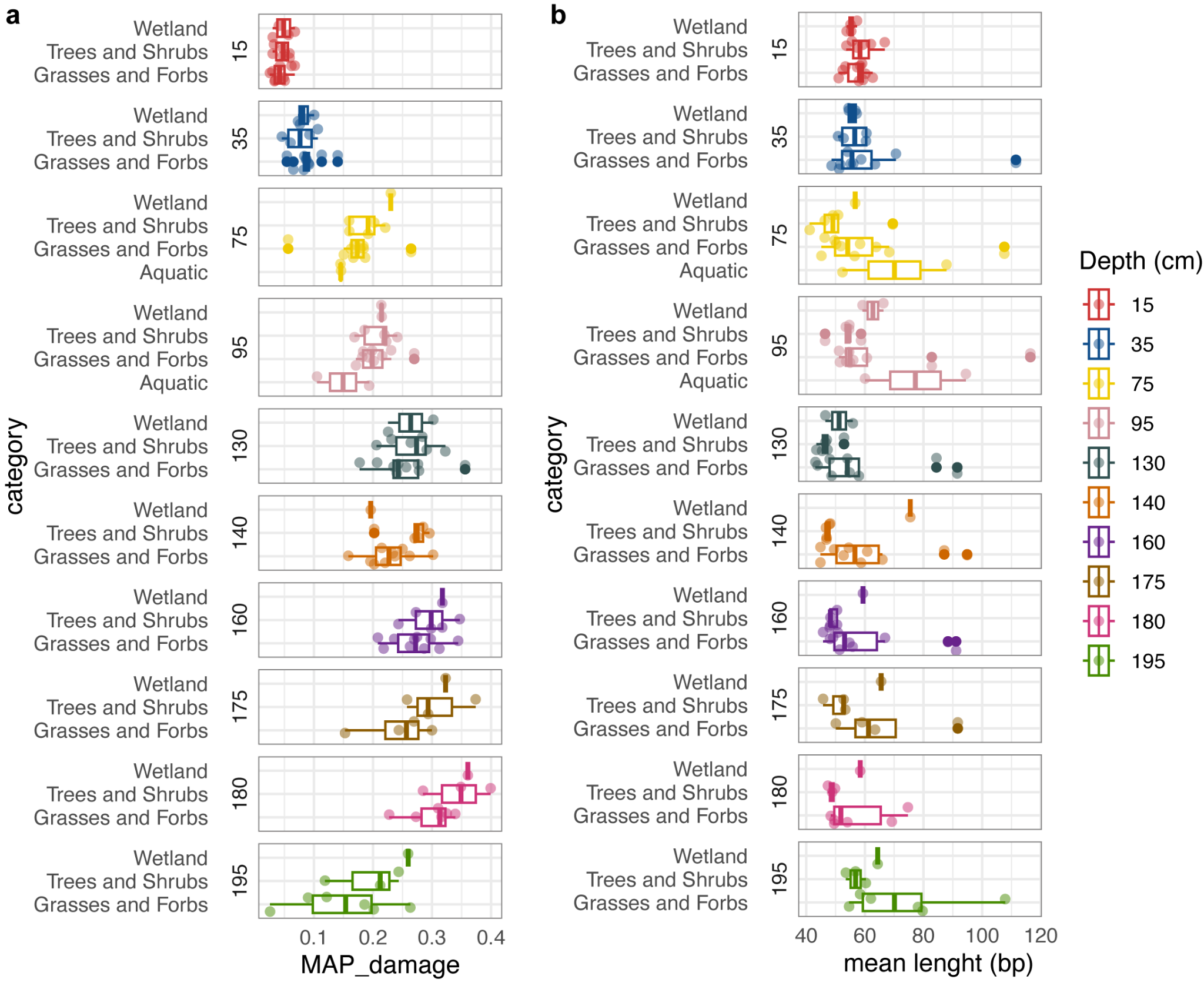
**

**Figure S4.10. Damage estimation (a) and mean length (b) of the most abundant plants at Magareći mlin.** The dataset was parsed using the depth-damage model (refer to Figure S4.4). Taxonomic categories are assigned at the family level, based on read counts above 500, and are consistent with those shown in Figure 5.

**
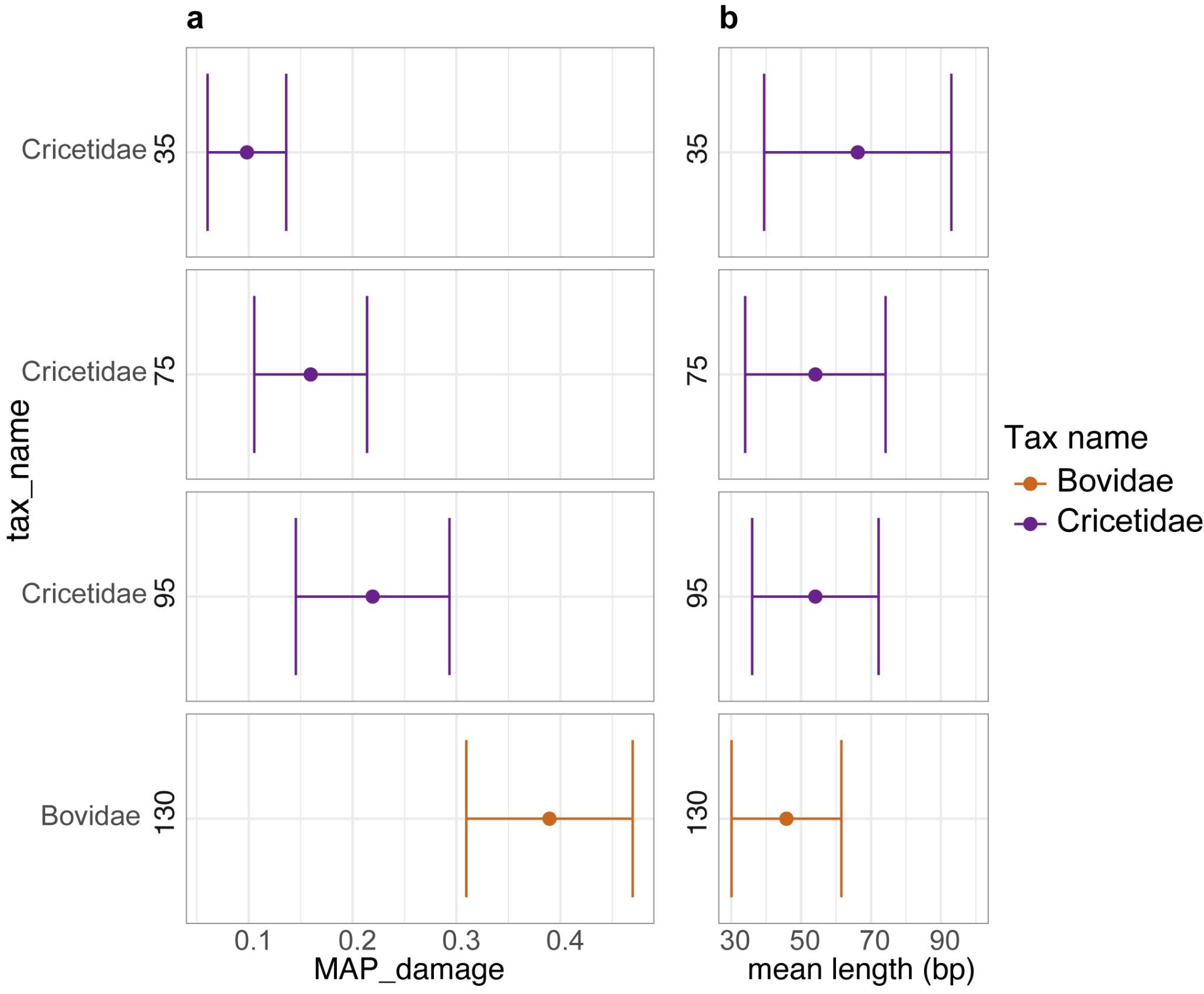
**

**Figure S4.11. Damage estimation (a) and mean length (b) of the most abundant animals at Magareći mlin.** The dataset was parsed using the depth-damage model (refer to Figure S4.4). Taxonomic categories are assigned at the family level, based on read counts above 100, and are consistent with those shown in Figure 5.

**
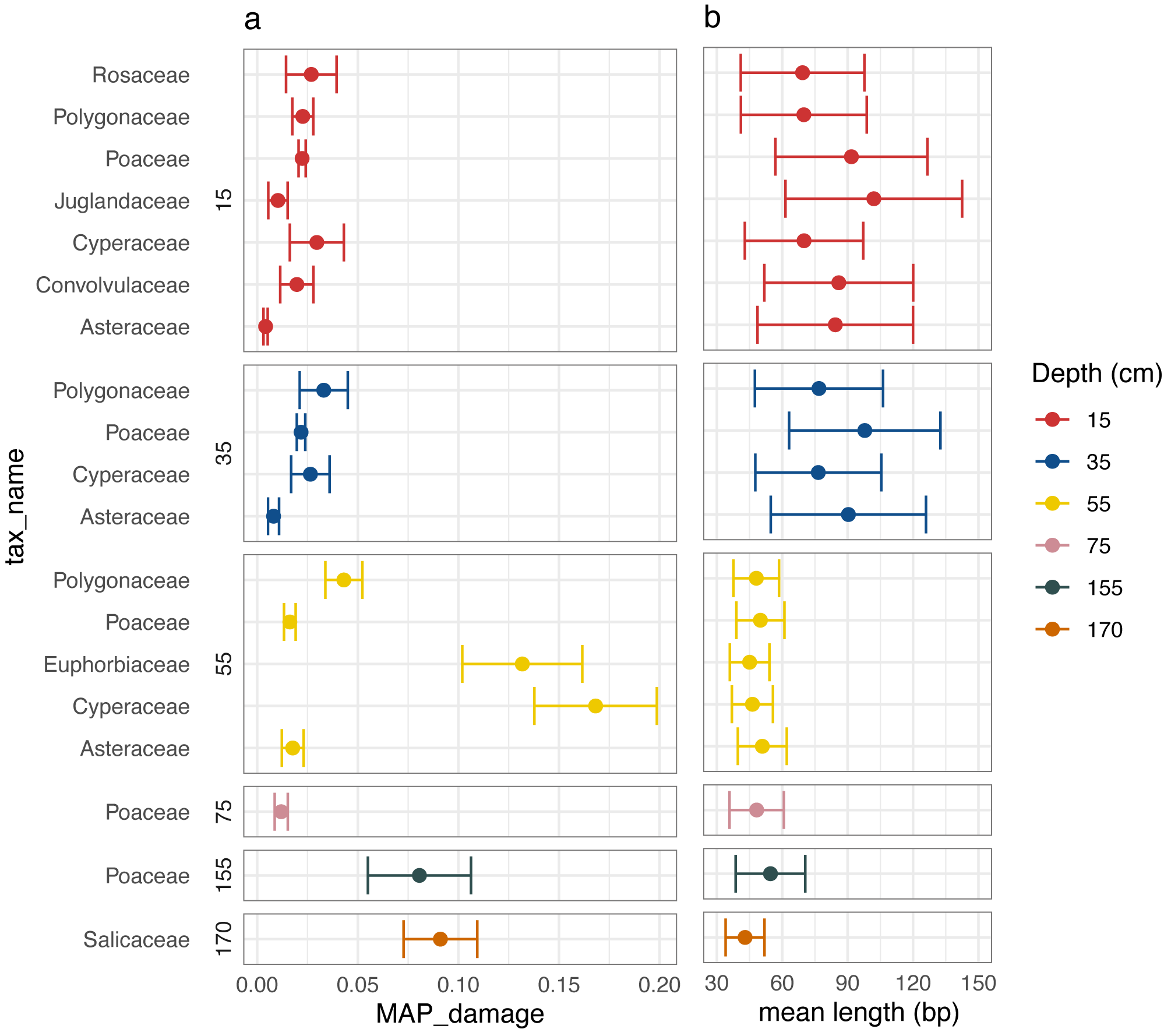
**

**Figure S4.12. Degree of damage (a) and mean length (b) of the most abundant plants at Starčevo-Grad (core 1).**

The dataset is shown at the family level based on read counts above 500 and a significance above 2.

**
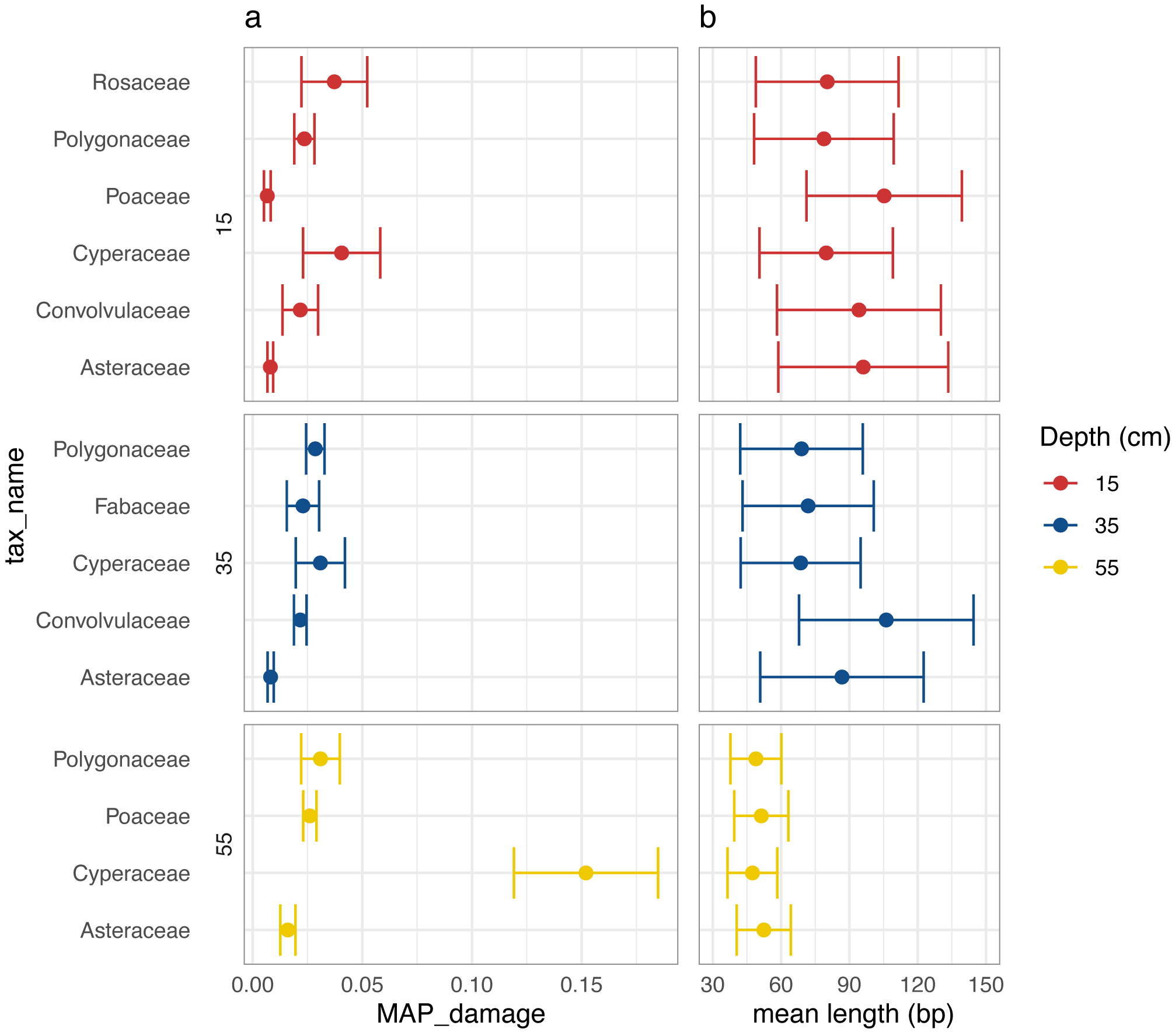
**

**Figure S4.13. Degree of damage (a) and mean length (b) of the most abundant plants at Starčevo-Grad (core 2).**

The dataset is shown at the family level based on read counts above 500 and a significance above 2.

**References**

1. [Ramsey CB. 2009 Bayesian Analysis of Radiocarbon Dates. *Radiocarbon* **51**, 337–360. (doi:](http://paperpile.com/b/dTR38M/tRQbF)[10.1017/S0033822200033865](http://dx.doi.org/10.1017/S0033822200033865)[)](http://paperpile.com/b/dTR38M/tRQbF)

2. [Reimer PJ *et al.* 2020 The IntCal20 Northern Hemisphere Radiocarbon Age Calibration Curve (0–55 cal kBP). *Radiocarbon* **62**, 725–757. (doi:](http://paperpile.com/b/dTR38M/jhWIW)[10.1017/RDC.2020.41](http://dx.doi.org/10.1017/RDC.2020.41)[)](http://paperpile.com/b/dTR38M/jhWIW)
